# Aerobic exercise prevents renal osteodystrophy via irisin-activated osteoblasts

**DOI:** 10.1172/jci.insight.184468

**Published:** 2025-01-30

**Authors:** Meng Wu, Huilan Li, Xiaoting Sun, Rongrong Zhong, Linli Cai, Ruibo Chen, Madiya Madeniyet, Kana Ren, Zhen Peng, Yujie Yang, Weiqin Chen, Yanling Tu, Miaoxin Lai, Jinxiu Deng, Yuting Wu, Shumin Zhao, Qingyan Ruan, Mei Rao, Sisi Xie, Ying Ye, Jianxin Wan

**Affiliations:** 1 Department of Nephrology, Blood Purification Research Center, the First Affiliated Hospital, Fujian Medical University, Fuzhou, China.; 2 Department of Nephrology, Longyan First Affiliated Hospital of Fujian Medical University, Longyan, China.; 3 Department of Nephrology, Xuanwu Hospital, Capital Medical University, Beijing, China.; 4 School of Pharmaceutical Science, Wenzhou Medical University, Wenzhou, China.; 5 Department of Cardiology, Basic Scientific Research Center, Longyan First Affiliated Hospital of Fujian Medical University, Longyan, China.; 6 Department of Cellular and Genetic Medicine, School of Basic Medical Sciences, Fudan University, Shanghai, China.; 7 Fundamental Research Center, Shanghai Yangzhi Rehabilitation Hospital (Shanghai Sunshine Rehabilitation Center), School of Medicine, Tongji University, Shanghai, China.; 8 Department of Oral Implantology, Stomatological Hospital and Dental School of Tongji University, Shanghai Engineering Research Center of Tooth Restoration and Regeneration, Shanghai, China.; 9 Fujian Clinical Research Center for Metabolic Chronic Kidney Disease, the First Affiliated Hospital, and; 10 Department of Nephrology, National Regional Medical Center, Binhai Campus of the First Affiliated Hospital, Fujian Medical University, Fuzhou, China.

**Keywords:** Bone biology, Cell biology, Metabolism, Bone disease

## Abstract

Renal osteodystrophy is commonly seen in patients with chronic kidney disease (CKD) due to disrupted mineral homeostasis. Given the impaired renal function in these patients, common antiresorptive agents, including bisphosphonates, must be used with caution or even contraindicated. Therefore, an alternative therapy without renal burden to combat renal osteodystrophy is urgently needed. Here, we report that clinically relevant aerobic exercise significantly prevents high-turnover renal osteodystrophy in CKD mice and patients with CKD without compromising renal function. Mechanistically, 4-week aerobic exercise in CKD mice increased expression of skeletal muscle PPARγ coactivator-1α (PGC-1α) and circulating irisin. Both exercise and irisin administration significantly activated osteoblasts, but not osteoclasts, via integrin αvβ5, thereby conferring bone quality benefits. Removal of irisin-influenced thermogenic adipose tissues or genetic ablation of uncoupling protein 1 did not alter the irisin-conferred antiosteodystrophy effect. Importantly, in a pilot clinical study, 12-week aerobic exercise in patients with high-grade CKD significantly increased circulating irisin and prevented osteodystrophy progression, without detectable renal burden. The combination of irisin and current antiresorptive agents effectively rescued renal osteodystrophy in mice. Our work provides mechanistic insights into the role of exercise and irisin in renal osteodystrophy, and it highlights a clinically relevant, low-cost, kidney-friendly therapy for patients with this devastating disease.

## Abstract 2

Aerobic exercise prevents renal osteodystrophy through irisin-activated osteoblasts, independent of adipose tissue browning.

## Graphical abstract

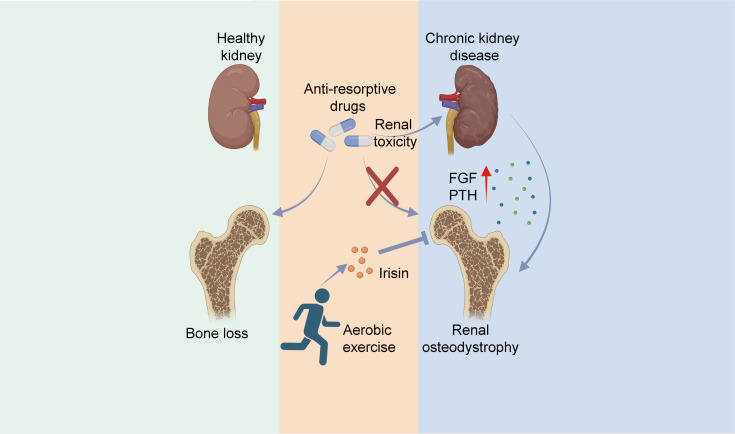

## Introduction

Chronic kidney disease (CKD), characterized by early insidiousness, long-term socioeconomic burden, and complex complications, is one of the most urgent global public health problems, with an estimated prevalence of 13.4% (1). Mineral homeostasis is often disturbed in patients with CKD due to altered calcium, phosphate, parathyroid hormone (PTH), FGF23, and vitamin D pathways. These changes in the bone/mineral axis are initiated by impaired renal function and lead to various clinical symptoms, including increased vascular calcification and high or low bone turnover osteodystrophy, which significantly reduce the quality of life and survival of patients with CKD (2, 3). Similar to osteoporosis in patients without CKD, renal osteodystrophy in patients with low-grade CKD can be managed with bisphosphonates, calcium supplements, parathyroid hormones, RANKL-neutralizing antibodies, and selective estrogen receptor modulators. However, renal osteodystrophy in patients with high-grade CKD has very limited medical options due to their severely impaired renal function. Bisphosphonates are contraindicated, and other drugs must be used cautiously after assessment of renal function (4). Therefore, an alternative therapy that does not jeopardize renal function is urgently needed for patients with CKD with renal osteodystrophy.

Clinically, lifestyle interventions are recommended for patients with CKD, including vitamin D supplementation, smoking cessation, alcohol restriction, and exercise (5, 6). Among these interventions, emerging evidence suggests that exercise improves renal osteodystrophy (7, 8). Several mechanisms have been proposed to underlie the antiosteoporotic effects of physical exercise, including FGF23 pathway (9), RANKL pathway (10), antiinflammatory effects (11), and IL-6 signaling (12). Although the majority of these studies focused on non-CKD diseases, research advances have suggested a similar muscle/bone axis in patients with CKD (13). In response to exercise, skeletal muscle produces various myokines that mediate muscle-organ crosstalk. Myokines and the muscle/bone axis have not been thoroughly investigated in renal osteodystrophy.

Irisin, discovered in 2012 by screening for myokines in response to PPARγ coactivator-1α (PGC-1α), is known to promote various healthy phenotypes including adipose tissue browning (14). In skeletal muscle cells, PGC-1α promotes the expression of the transmembrane protein fibronectin type III domain containing 5 (FNDC5), which is further truncated and the extracellular portion is released as irisin (15). Irisin has 100% sequence homology between humans and mice. In humans, FNDC5 is highly expressed in skeletal muscle, heart, tongue, and rectum. In mice, the majority of circulating irisin is derived from muscle secretion (16). Various types of exercise, including short-term high-intensity exercise, long-term endurance training, and strength training, can increase irisin secretion (17). In addition to adipose tissue browning and metabolic diseases, a role for irisin in maintaining bone homeostasis has been proposed (18). Irisin promotes osteoblast differentiation, possibly via the αvβ5 integrin receptor (19), while its effect on osteoclasts is not clear. Of note, the role of irisin in CKD-associated bone disorders has not been investigated.

In this work, using a clinically relevant 4-week aerobic exercise regimen in CKD mouse models, we demonstrate that aerobic exercise induces circulating irisin and activates osteoblasts in CKD mice, thereby ameliorating osteodystrophy. Daily administration of irisin to mimic daily aerobic exercise induced proosteogenic effects in CKD mice. Irisin-induced antiosteodystrophy effects are independent of adipose tissue browning but dependent on direct irisin-osteoblast interaction. Compared with osteoclasts, osteoblasts had a significantly higher expression of αvβ5 integrin, conferring exercise-induced bone quality benefits. Twelve-week aerobic exercise in patients with CKD significantly increased circulating irisin and prevented the progression of renal osteodystrophy, with no detectable renal burden. This study provides the mechanistic insights into exercise-alleviated renal osteodystrophy and advocates exercise or irisin administration as clinically relevant, low-cost, kidney-friendly therapeutic options for renal osteodystrophy.

## Results

### Aerobic exercise ameliorates renal osteodystrophy in CKD mice.

To test the effect of aerobic exercise on renal osteodystrophy, a CKD mouse model was established by 5/6 nephrectomy in 8-week-old mice. Six weeks after surgery, renal function was significantly impaired, as evidenced by robust increases in the renal function markers creatinine, urea nitrogen, and uric acid compared with those from sham-operated control mice (Supplemental Figure 1, A and B; supplemental material available online with this article; https://doi.org/10.1172/jci.insight.184468DS1), suggesting the establishment of CKD. To test whether this model instigates renal osteodystrophy, μ-CT was performed on mouse femurs. Measurements confirmed significantly reduced total bone volume fraction (BV/TV) and trabecular number (Tb.N) as well as robustly increased trabecular spacing (Tb.Sp) and structure model index (SMI), compared with femurs from sham-operated control mice (Supplemental Figure 1C). Femur histology confirmed a profound osteodystrophy phenotype in CKD mice (Supplemental Figure 1D). These results indicate renal osteodystrophy after 6 weeks of CKD in mice. Therefore, we used 6 weeks after modeling as the time point to study renal osteodystrophy afterward.

**Figure 1:**
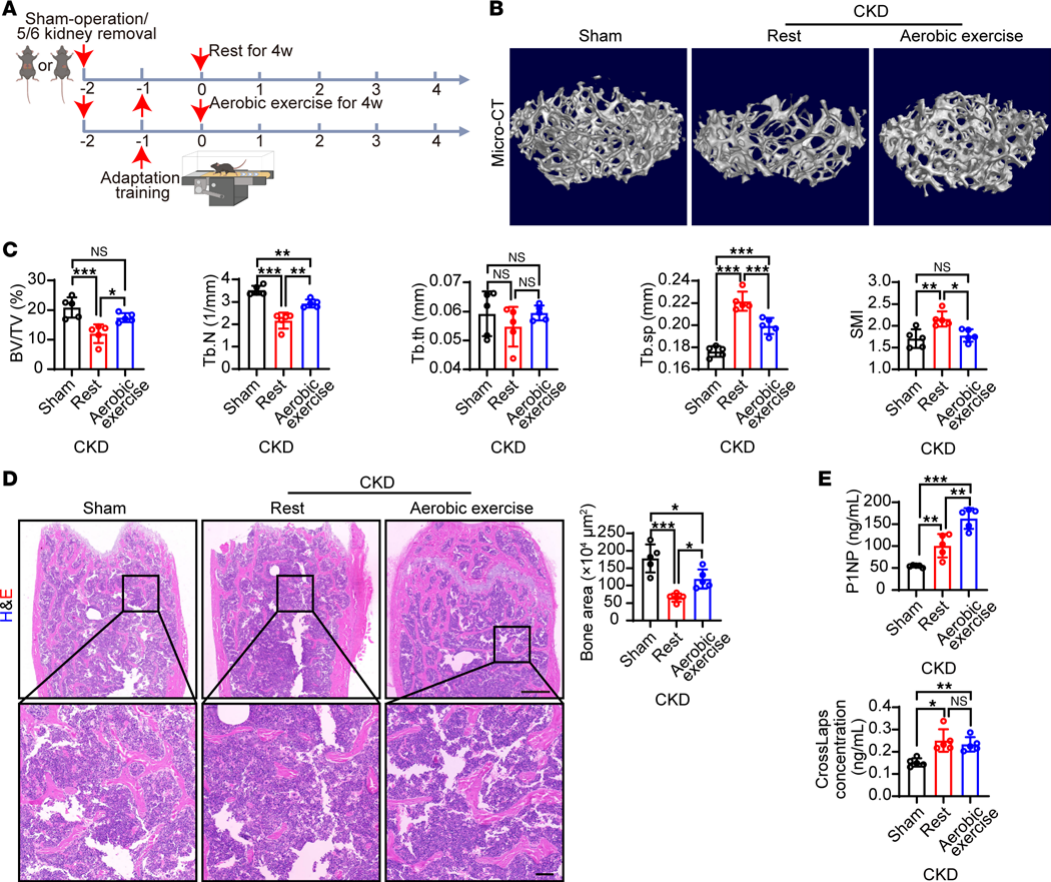
Aerobic exercise ameliorates renal osteodystrophy in CKD mice. (**A**) Schematic diagram of the CKD model establishment and exercise regimen. Nephrectomy of left kidney and two-thirds of right kidney was performed for CKD modeling. Sham-operated mice were used as controls. One week after surgery, exercise training was performed by 1 week of adaptation and 4 weeks of treadmill aerobic exercise. (**B** and **C**) Representative μ-CT images of the femur tissues from resting or exercise-trained CKD mice. Sham-operated mice served as controls. Analysis of BV/TV, Tb.N, trabecular thickness (Tb.th), Tb.Sp, and SMI of these groups (*n* = 5 mice per group). (**D**) Representative H&E staining images of the femur tissues from resting or exercise-trained CKD mice. Sham-operated mice served as controls. Scale bar: 500 μm (upper panel), 100 μm (lower panel). Quantification of the bone area (*n* = 5 mice per group). (**E**) P1NP and crosslaps in serum from resting or exercise-trained CKD mice. Sham-operated mice served as controls (*n* = 5 mice per group). Data were analyzed by 1-way ANOVA (**C**–**E**). *P < 0.05; **P < 0.01; ***P < 0.001. Data were presented as mean ± SD.

To study the effect of physical exercise on renal osteodystrophy, we chose a light aerobic exercise scheme because of its known cardiovascular and musculoskeletal benefits in humans and because it is less challenging for older adults and patients. A well-accepted light aerobic exercise regime was applied in CKD mice (20). After 1 week of adaptive training, mice were randomly assigned to a 4-week aerobic exercise group or a resting group (Figure 1A). Light aerobic exercise did not alter bodyweight or average food/water intake in CKD mice (Supplemental Figure 1, E and F). At the end of 4 weeks of aerobic exercise, mice were sacrificed and bone homeostasis was determined. Interestingly, 4-week aerobic exercise significantly improved BV/TV and Tb.N, and reduced Tb.Sp and SMI, compared with femurs from resting CKD mice (Figure 1, B and C). Femur histology confirmed the preventive effect of aerobic exercise on renal osteodystrophy (Figure 1D). Markers of bone homeostasis were examined. The bone formation marker procollagen type I N-terminal propeptide (P1NP) was increased in CKD mice and was further markedly upregulated by exercise, while the osteoclast marker crosslaps remained at high levels (Figure 1E), suggesting that exercise stimulates osteogenesis in these mice.

The increased bone formation marker and osteoclast marker suggested that the CKD mice might have a high bone turnover subtype of renal osteodystrophy, which is characterized by increased bone resorption and formation rates. Elevated PTH levels are important in the pathogenesis of these states, and FGF23 plays a central role in regulating PTH levels (21). We therefore assessed serum PTH and FGF23 levels as well as bone histomorphometry in this model. As expected, CKD mice had markedly elevated PTH and FGF23 levels (Supplemental Figure 2, A and B), suggesting the high bone turnover subtype of renal osteodystrophy in this model. Trabeculae were significantly reduced, and Trap staining showed that the number of osteoclasts around trabeculae was significantly increased in CKD mice (Supplemental Figure 2, C and D). Interestingly, although exercise prevented trabecular loss, it did not alter serum PTH and FGF23 levels or the number of osteoclasts around trabeculae (Supplemental Figure 2, A–D), suggesting that exercise does not alter the subtype of renal osteodystrophy. Together, these results indicate that light aerobic exercise ameliorates renal osteodystrophy in mice.

### Aerobic exercise increases circulating irisin via PGC-1α in CKD mice.

**Figure 2:**
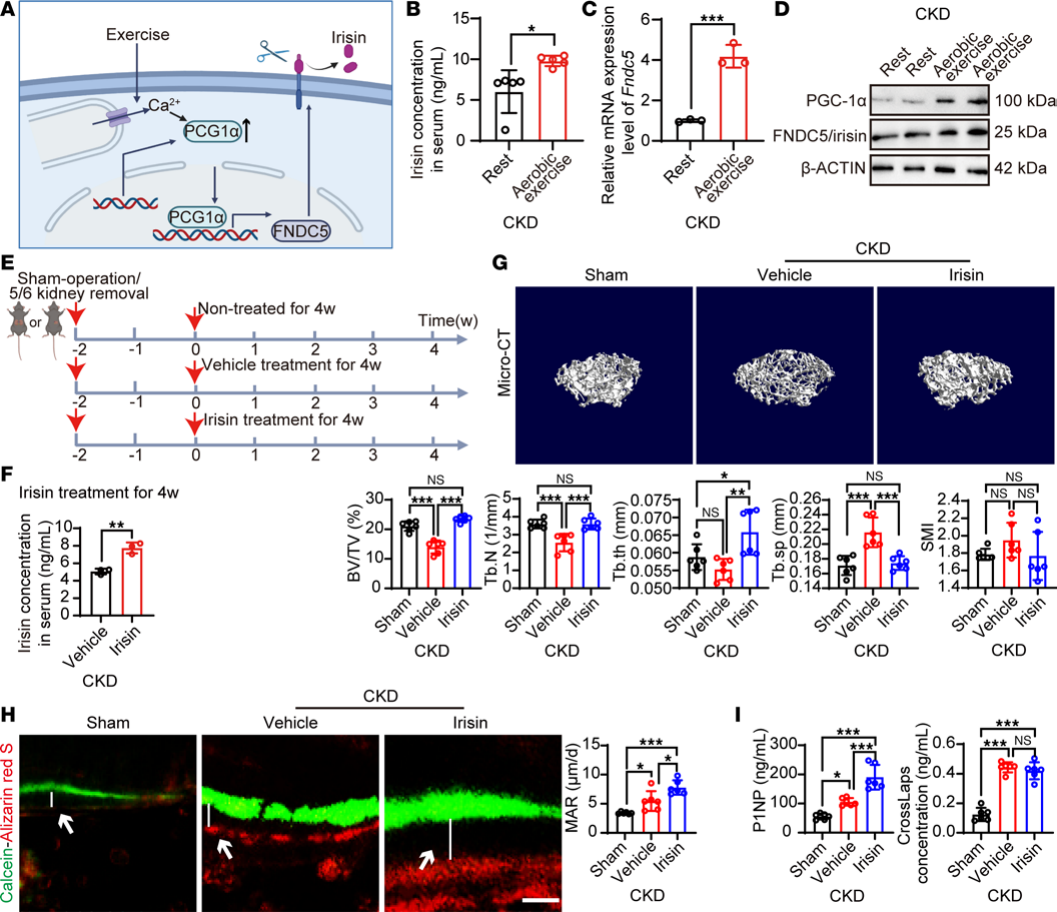
Aerobic exercise/PGC-1α/irisin axis prevents renal osteodystrophy in mice. (**A**) Diagram of the exercise-induced irisin release. Exercise induces transcription of PGC-1α and FNDC5 in skeletal muscle. The transmembrane protein FNDC5 can be proteolytically cleaved and secreted as circulating irisin. (**B** and **C**) Circulating irisin levels in serum from resting or exercise-trained CKD mice (*n* = 5 mice per group). *Fndc5* RNA expression levels in skeletal muscle tissues from resting or exercise-trained CKD mice (*n* = 3 mice per group). (**D**) Western blot of PGC-1α and FNDC5/irisin in skeletal muscle tissues from resting or exercise-trained CKD mice. β-Actin served as controls. (**E**) Schematic diagram of irisin administration in CKD model. (**F**) Circulating irisin levels in serum from vehicle- or irisin-treated CKD mice (*n* = 3 mice per group). (**G**) Representative μ-CT images of the femur tissues from vehicle- or irisin-treated CKD mice. Sham-operated mice served as controls. Analysis of BV/TV, Tb.N, trabecular thickness (Tb.th), Tb.Sp, and SMI of these groups (*n* = 6 mice per group). (**H**) Representative images of Calcein–Alizarin red S double labeling of femurs from vehicle- or irisin-treated CKD mice. Quantification of mineral apposition rate (MAR) (*n* = 6 mice per group). Arrows: mineralized distance. (**I**) P1NP and crosslaps in serum from vehicle- or irisin-treated CKD mice. Sham-operated mice served as controls (*n* = 6 mice per group). Data were analyzed by unpaired, 2-tailed Student’s *t* test (**B**, **C**, and **F**) and 1-way ANOVA (**G**–**I**). *P < 0.05; **P < 0.01; ***P < 0.001. Data were presented as mean ± SD.

The molecular mechanisms of physical activity in the prevention of bone loss remain elusive. The well-recognized muscle-bone crosstalk mechanisms are mainly mediated by muscle-secreted factors such as cytokines, exosomes, and myokines. Among this exercise-released secretome, irisin is expressed and released by myocytes and may mediate the muscle/bone axis (Figure 2A). Although the physiological functions of irisin have been described in rodents and humans (22), the role of irisin in pathological conditions such as renal osteodystrophy is not fully understood. To test whether irisin is involved in renal osteodystrophy, circulating irisin was measured by ELISA. As expected, 4-week aerobic exercise upregulated circulating irisin in CKD mice to approximately 2-fold (Figure 2B). In muscle, exercise strongly upregulated the *Fndc5* mRNA expression (Figure 2C). Exercise-linked FNDC5/irisin is reported to be regulated by PGC-1α. Indeed, aerobic exercise robustly increased skeletal muscle PGC-1α expression and FNDC5/irisin expression in CKD mice (Figure 2D). Of note, immunological methods for detecting irisin inevitably detect its precursor FNDC5 in tissues; therefore, the term “FNDC5/irisin” was used for tissue detection and the term “irisin” for serum detection. These results were consistent with those reported in non-CKD healthy animals, suggesting a similar exercise-induced PGC-1α/FNDC5/irisin axis in CKD mice.

### Irisin administration prevents renal osteodystrophy in mice.

To investigate whether the antiosteodystrophy effect of exercise is mediated by irisin, we injected irisin 100 μg/kg per day i.p. for 4 weeks (Figure 2E). Interestingly, irisin administration significantly upregulated circulating irisin levels (Figure 2F), improved BV/TV and Tb.N, and reduced Tb.Sp and SMI, compared with femurs from vehicle-treated CKD mice (Figure 2G). We next performed histology and histomorphometry in these mice. Femur histology confirmed the preventive effect of irisin on renal osteodystrophy (Supplemental Figure 3A). Trabeculae loss was rescued by irisin administration (Supplemental Figure 3A). Similar to exercise training, irisin did not alter serum PTH and FGF23 levels or the number of osteoclasts around trabeculae (Supplemental Figure 3, A–C). We also performed dynamic histomorphometry by injecting with Calcein and Alizarin red S on day 22 and day 25 of the 4-week irisin administration. After irisin administration, bone formation activity was increased in the CKD mice, and irisin administration further increased the bone formation activity compared with the vehicle-treated group (Figure 2H). Serum marker P1NP was significantly stimulated by irisin stimulation, while the osteoclast marker crosslaps remained at high level, reproducing the effect of aerobic exercise in CKD mice (Figure 2I). These results suggest that irisin may alleviate high bone turnover renal osteodystrophy but has no effect on bone resorption. To validate this hypothesis, blood phosphorus and calcium levels were measured and found to be unaltered by irisin administration (Supplemental Figure 3D), suggesting an insignificant effect of irisin on bone resorption.

### Sustained irisin administration is required to prevent renal osteodystrophy.

**Figure 3:**
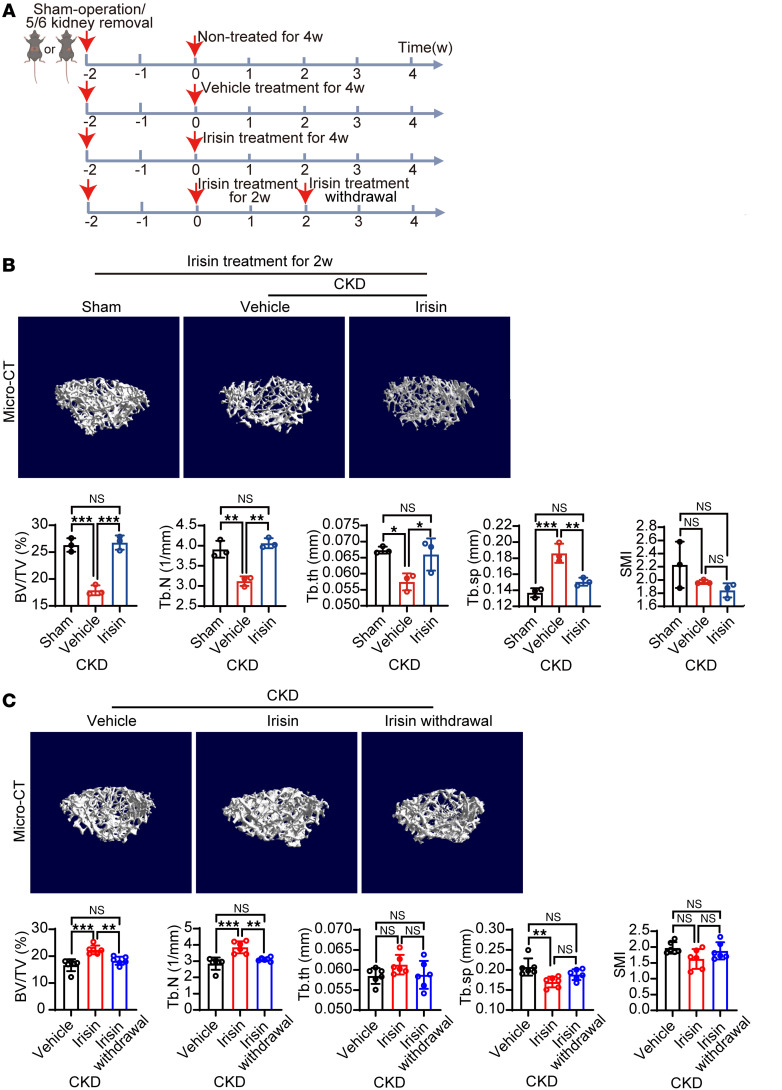
Sustained irisin administration is required to prevent renal osteodystrophy. (**A**) Schematic diagram of irisin administration and irisin withdrawal regimen in CKD model. (**B**) Representative μ-CT images of the femur tissues from 2-week vehicle- or irisin-treated CKD mice. Sham-operated mice served as controls. Analysis of BV/TV, Tb.N, trabecular thickness (Tb.th), Tb.Sp, and SMI of these groups (*n* = 3 mice per group). (**C**) Representative μ-CT images of the femur tissues from CKD mice treated with 4-week vehicle, 4-week irisin, or irisin withdrawal (2-week irisin and 2-week vehicle). Analysis of BV/TV, Tb.N, trabecular thickness (Tb.th), Tb.Sp, and SMI of these groups (*n* = 6 mice per group). Data were analyzed by 1-way ANOVA (**B** and **C**). *P < 0.05; **P < 0.01; ***P < 0.001. Data were presented as mean ± SD.

To investigate whether the preventive effect of irisin is sustained, we examined the bone parameters at week 2 of irisin administration (Figure 3A). As expected, 2-week irisin administration maintained bone volume and trabecular number (Figure 3B), similar to those of the sham-operated group and those of the 4-week treatment group (Figure 2G). Subsequently, withdrawal experiments were conducted after 2 weeks of treatment (Figure 3A). Intriguingly, irisin withdrawal in CKD mice for 2 weeks resulted in low bone mineral density (BMD) in CKD mice similar to vehicle-treated CKD mice (Figure 3C), underscoring the necessity for continuous irisin therapy to prevent renal osteodystrophy. Together, these results indicate that aerobic exercise–induced irisin secretion prevents renal osteodystrophy, and that sustained irisin therapy should be used.

### The antirenal osteodystrophy effect of irisin is independent of adipose tissue browning.

**Figure 4:**
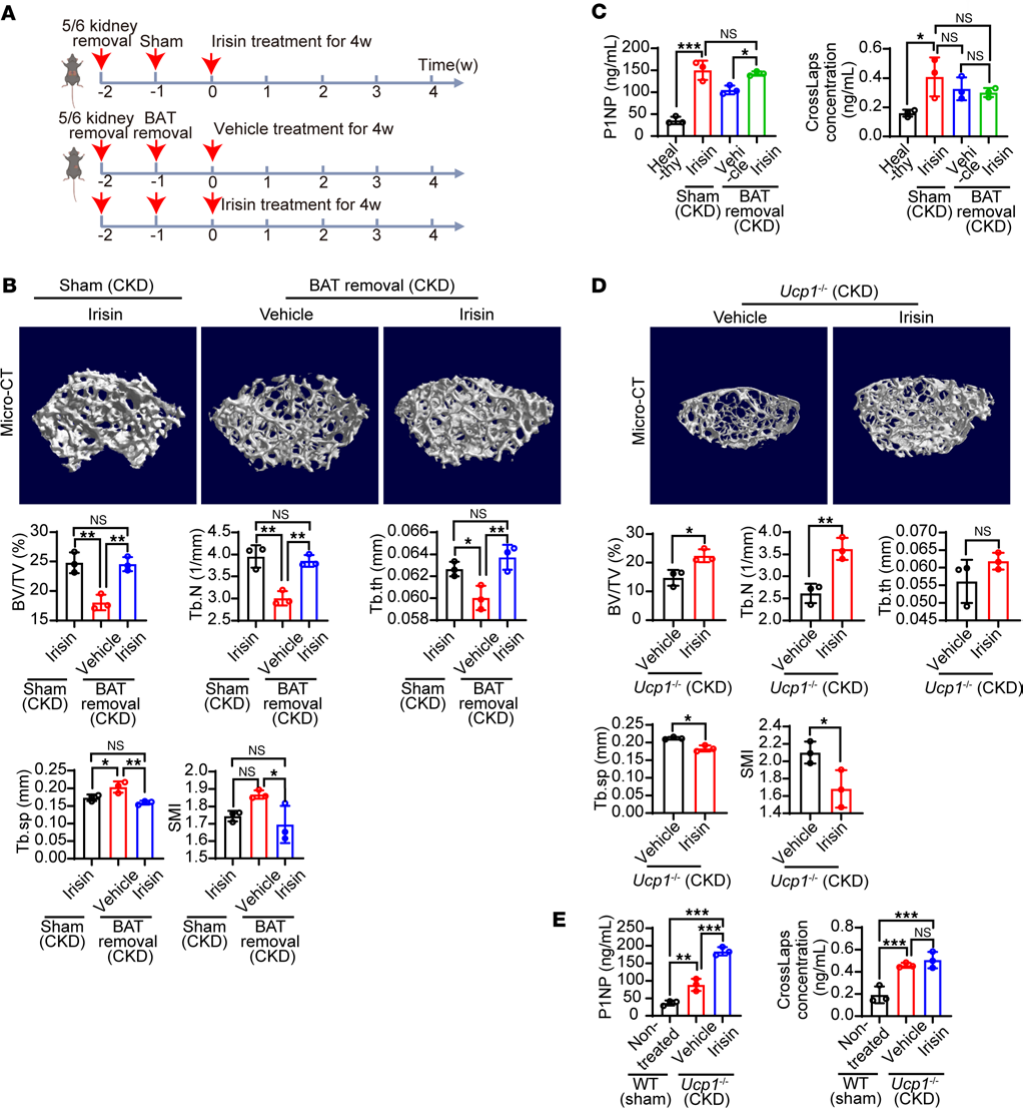
Adipose tissue browning–independent antiosteodystrophy effect of irisin in CKD mice. (**A**) Schematic diagram of BAT removal and irisin administration regimen in CKD model. (**B**) Representative μ-CT images of the femur tissues from vehicle- or irisin-treated BAT-removed CKD mice. Irisin-treated sham-operated CKD mice served as controls. Analysis of BV/TV, Tb.N, trabecular thickness (Tb.th), Tb.Sp, and SMI of these groups (*n* = 3 mice per group). (**C**) P1NP and crosslaps in serum from vehicle- or irisin-treated BAT removed CKD mice. Healthy mice and irisin-treated sham-operated CKD mice served as controls (*n* = 3 mice per group). (**D**) Representative μ-CT images of the femur tissues from WT or *Ucp1^–/–^* CKD mice. Analysis of BV/TV, Tb.N, Tb.th, Tb.Sp, and SMI of these groups (*n* = 3 mice per group). (**E**) P1NP and crosslaps in serum from WT or *Ucp1^–/–^* CKD mice (*n* = 3 mice per group). Data were analyzed by 1-way ANOVA (**B**, **C**, and **E**) and unpaired, 2-tailed Student’s *t* test (**D**). *P < 0.05; **P < 0.01; ***P < 0.001. Data were presented as mean ± SD.

Since the original report describing this myokine, irisin has been recognized as a hormone that triggers browning of white adipose tissue (WAT) and activation of brown adipose tissue (BAT) (14). Several studies support that the activation of adipose tissue, particularly BAT, is associated with a healthy phenotype, including healthy bone metabolism (23–25), as an adipose tissue/bone axis. To exclude this possibility, we performed experiments to surgically remove BAT in CKD mice (Figure 4A). Interestingly, BAT removal did not alter the irisin-induced bone benefits, as evident by BV/TV and other parameters (Figure 4B). Femur histology confirmed no change in irisin-treated renal osteodystrophy with BAT removal (Supplemental Figure 4A). Similarly, irisin-rescued P1NP was observed in mice with BAT removal (Figure 4C). These results suggest that the antiosteodystrophy effect of irisin is independent of BAT.

To further validate this result, we used uncoupling protein 1^–/–^ (*Ucp1^−/−^*)mice, in which UCP1, the key mitochondrial protein responsible for adipose tissue browning, is knocked out, to exclude the involvement of adipose tissue browning in the irisin-associated antiosteodystrophy effect. As expected, deletion of the *Ucp1* gene did not abolish the antiosteodystrophy effect of irisin in CKD model (Figure 4, D and E). μ-CT, femur histology, and serum P1NP and crosslaps showed a similar trend in both irisin-treated WT CKD mice and in irisin-treated *Ucp1^−/−^* CKD mice (Figure 2; Figure 4, D and E; and Supplemental Figure 4B). Taken together, these results suggest that the antirenal osteodystrophy effect of irisin is independent of the adipose tissue/bone axis.

### Direct irisin/integrin αvβ5/osteoblast axis prevents renal osteodystrophy.

**Figure 5:**
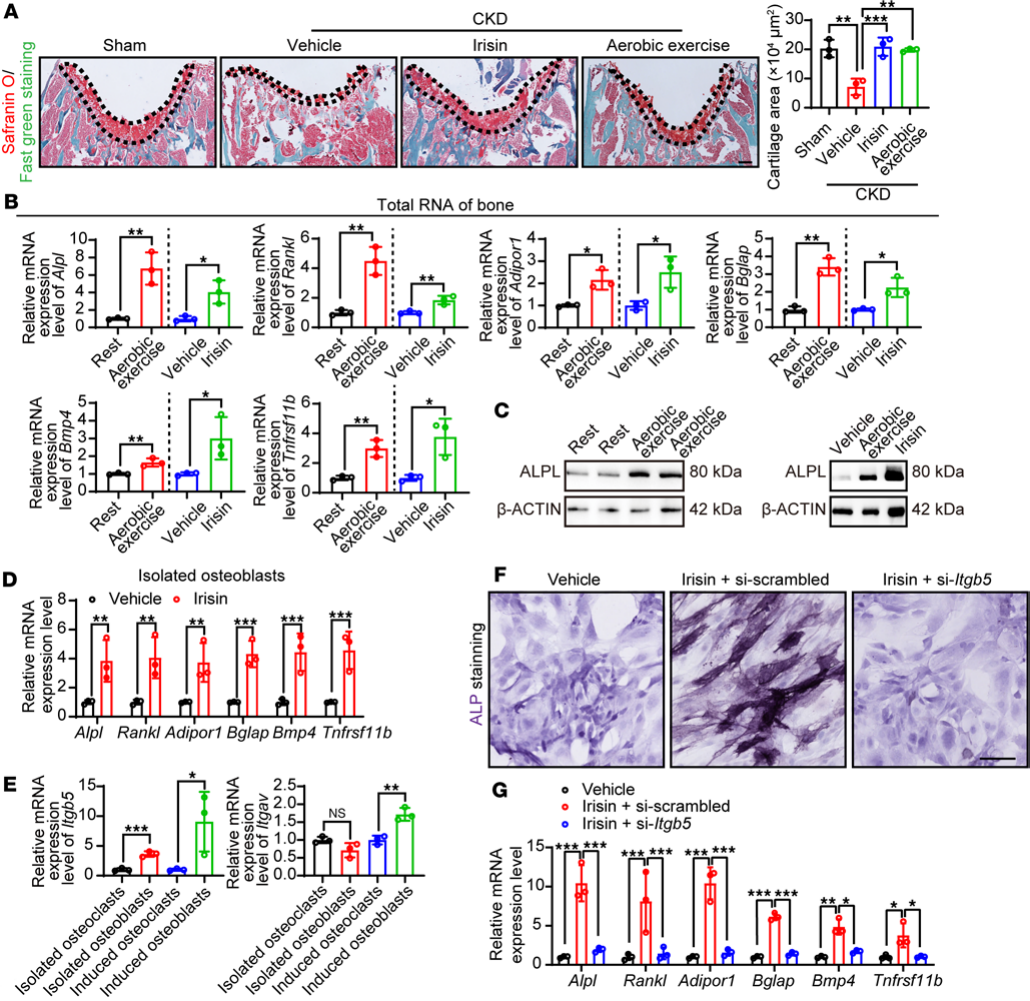
Irisin/integrin αvβ5/osteoblast axis prevents renal osteodystrophy. (**A**) Representative Safranin O/Fast green staining images of the femur tissues from vehicle- or irisin-treated CKD mice. Sham-operated mice and exercise-trained CKD mice served as controls. Scale bar: 100 μm. Quantification of the bone area (*n* = 3 mice per group). (**B**) *Alpl*, *Rankl*, *Bglap*, *Adipor1*, *Bmp4*, and *Tnfrsf11b* RNA expression levels in femur bone tissues from various groups (*n* = 3 mice per group). (**C**) Western blot of ALPL in femur bone tissues from various groups. β-Actin served as controls. (**D**) *Alpl*, *Rankl*, *Bglap*, *Adipor1*, *Bmp4*, and *Tnfrsf11b* RNA expression levels in RANK^–^CD45^–^ osteoblast cell populations isolated from femur bone tissues from vehicle- or irisin-treated CKD mice (*n* = 3 mice per group). (**E**) *Itgav* and *Itgb5* RNA expression levels in freshly isolated and differentiated osteoblasts and osteoclasts. (**F**) Representative alkaline phosphatase staining images of irisin administrated MC3T3-E1 differentiated osteoblasts pretreated with si-scrambled or si-*Itgb5*. (**G**) *Alpl*, *Rankl*, *Bglap*, *Adipor1*, *Bmp4*, and *Tnfrsf11b* RNA expression levels in various groups of MC3T3-E1 differentiated osteoblasts (*n* = 3 mice per group). Data were analyzed by 1-way ANOVA (**A**, **E**, and **G**) and unpaired 2-tailed Student’s *t* test (**B** and **D**). *P < 0.05; **P < 0.01; ***P < 0.001. Data were presented as mean ± SD.

Knowing that adipose tissue is not involved in the irisin-prevented osteodystrophy, we then focused on the direct irisin/bone axis. To study the target cell type of irisin, histochemical analysis of Safranin O and Fast Green was performed in femurs from sham-operated mice, CKD mice, irisin-treated CKD mice, and aerobic exercise–trained CKD mice. Histochemistry revealed a significant increase in cartilage area in both irisin-treated and aerobic exercise–trained groups compared with the nontreated CKD group (Figure 5A), suggesting that irisin prevents cartilage loss in CKD mice. Femur bone tissue was dissected and total RNA was harvested for quantitative PCR (qPCR) analysis. Interestingly, osteoblast activation markers, including alkaline phosphatase, liver/bone/kidney (*Alpl*), TNF superfamily member 11 (*Rankl*), adiponectin receptor 1 (*Adipor1*), bone γ carboxyglutamate protein (*Bglap*), *Bmp4*, and TNF receptor superfamily, member 11b (*osteoprotegerin, Tnfrsf11b*) were markedly upregulated by irisin or aerobic exercise (Figure 5B). In contrast, osteoclast markers such as cathepsin K (*Ctsk*), acid phosphatase 5 (*Acp5*), and matrix metallopeptidase 9 (*Mmp9*) remained unaltered by irisin or aerobic exercise (Supplemental Figure 5A), suggesting an osteogenesis in these bone tissues. As a representative gene, ALPL protein expression was validated (Figure 5C). To investigate the cellular response to irisin in vivo, we isolated fresh osteoblasts and osteoclasts from femur tissues (26, 27). Interestingly, in freshly isolated osteoblasts, a selected panel of osteoblast activation markers were dramatically upregulated up to approximately 4-fold upon irisin treatment (Figure 5D), whereas osteoclasts were unaltered (Supplemental Figure 5B), suggesting that irisin may directly activate osteoblasts in CKD mice. Reports have suggested integrin αvβ5 as a potential receptor for irisin (28). We further investigated the expression of this receptor in isolated fresh osteoblasts/osteoclasts and differentiated osteoblasts/osteoclasts. Although Integrin subunit α V (*Itgav*) was similarly expressed in both osteoblasts and osteoclasts, Integrin subunit β 5 (*Itgb5*) expression was significantly higher in osteoblasts than in osteoclasts (Figure 5E), suggesting that irisin acts on osteoblasts rather than osteoclasts. We next validated this pathway by loss-of-function experiments using siRNA knockdown of *Itgb5* in an in vitro osteoblast differentiation assay (Supplemental Figure 5C). As expected, knockdown of *Itgb5* significantly reduced the irisin-induced osteoblast differentiation and mineralization, as shown by alkaline phosphatase staining, Alizarin red S staining, and osteoblast marker expression levels (Figure 5, F and G, and Supplemental Figure 5D). Together, these results suggest a direct irisin/integrin αvβ5/osteoblast axis in CKD mice.

### Aerobic exercise increases circulating irisin and prevents renal osteodystrophy in patients.

**Figure 6:**
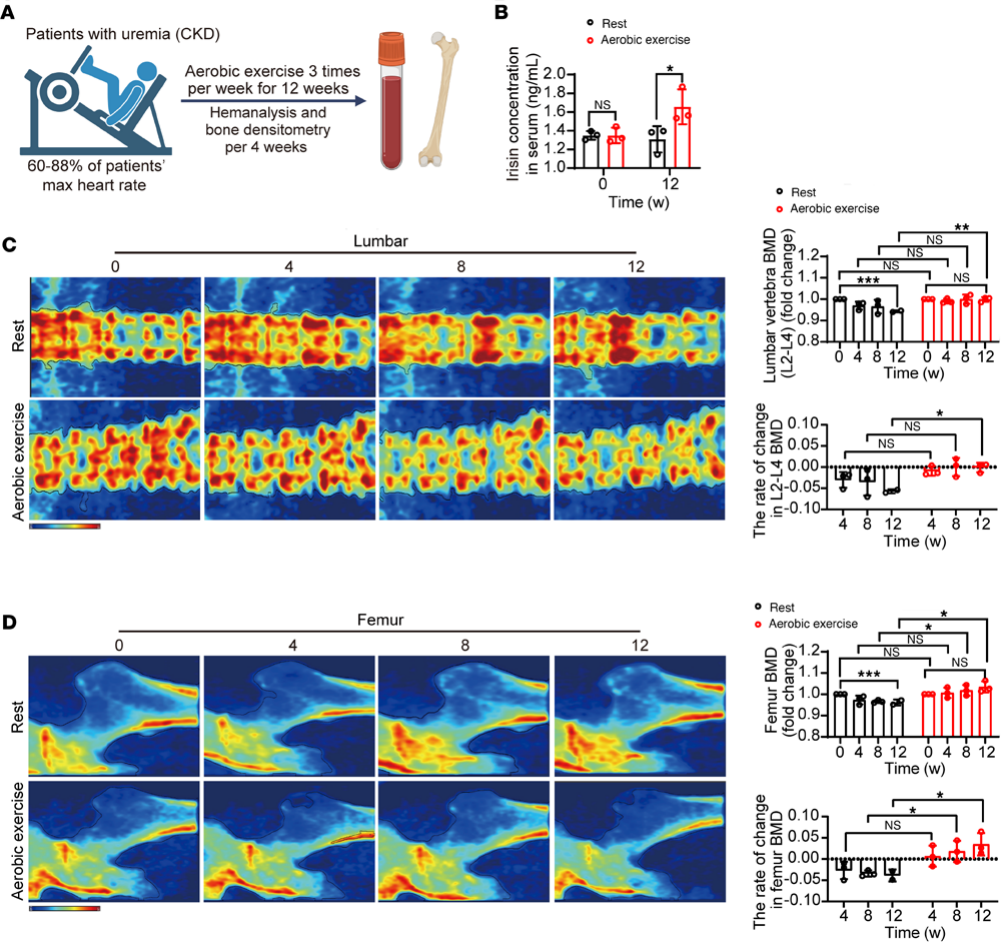
Aerobic exercise increases circulating irisin and prevents renal osteodystrophy in patients. (**A**) Schematic diagram of patients with ESRD receiving intradialysis light aerobic exercise training (*n* = 3 patients per group) or a nonexercise lifestyle (*n* = 3 patients per group) for 12 weeks. An adjustable, bed-mounted treadmill was used as the training method. (**B**) Serum circulating irisin levels in resting or exercise-trained patients with ESRD at week 0 and week 12 (*n* = 3 patients per group). (**C**) Representative dual-energy x-ray absorptiometry images of lumbar in resting or exercise-trained patients. Red: area of osteodystrophy. Quantification of L2-L4 vertebral BMD and BMD change rate in resting or exercise-trained patients at week 0, 4, 8, 12 (*n* = 3 patients per group). (**D**) Representative dual-energy x-ray absorptiometry images of femur in resting or exercise-trained patients. Red: area of osteodystrophy. Quantification of L2-L4 femoral BMD and BMD change rate in resting or exercise-trained patients at week 0, 4, 8, 12 (*n* = 3 patients per group). Data were analyzed by 2-way ANOVA (**B**–**D**). *P < 0.05; **P < 0.01; ***P < 0.001. Data were presented as mean ± SD.

To investigate whether this mechanism of the antirenal osteodystrophy effect of aerobic exercise can be translated in patients with end-stage renal disease (ESRD), we conducted a prospective pilot clinical study in 6 patients with ESRD receiving dialysis 3 times per week. Patients with ESRD were randomized to intradialysis light aerobic exercise (*n* = 3) or a nonexercise lifestyle (*n* = 3) for 12 weeks (Figure 6A). Progressive lower limb resistance training appears to be the most effective type of exercise intervention for BMD at the femoral neck (29). Therefore, we chose an adjustable, bed-mounted treadmill as the training method and used an established aerobic exercise regime for these patients (30). Baseline characteristics were comparable in terms of age, sex, or clinical stage (Supplemental Table 1). Inclusion criteria were strictly followed as described in the Methods section. During the study, participants were closely monitored for possible and unintended adverse events by the study team and an experienced care team. No adverse events were observed in any of the participants during the study.

Compared with healthy volunteers, these 6 patients with ESRD have significantly elevated serum PTH and FGF23 levels (Supplemental Figure 6, A and B), suggesting the high bone turnover renal osteodystrophy. After 12 weeks of exercise, similar to what we observed in animal models, switching to light aerobic exercise did not alter the participants’ bodyweight (Supplemental Figure 6C) or kidney function (Supplemental Figure 6D), but it significantly increased circulating irisin levels (Figure 6B). Of note, the increase of irisin was approximately 1.3-fold. These results were consistent with previous reports (31). Importantly, L2-L4 vertebral BMD showed a trend of decrease in the resting group, suggesting renal osteodystrophy progression (Figure 6C). In contrast, L2-L4 vertebral BMD in the aerobic exercise–trained group remained unchanged (Figure 6C). At week 0, there was no significant difference in BMD between these 2 groups, whereas at week 12, there was a significant BMD change in the exercised patients with ESRD, compared with the resting patients with ESRD (Figure 6C). Similar results were observed for femur BMD, as a significant increase was observed from week 8 of exercise (Figure 6D), demonstrating the preventive effect of aerobic exercise on renal osteodystrophy. We then examined blood calcium and phosphorus in these 2 ESRD groups and observed an increase of blood phosphorus in the resting group, while in aerobic exercise–trained patients with ESRD, blood phosphorus levels were unaltered (Supplemental Figure 6E). Notably, no changes in blood calcium were observed in either group (Supplemental Figure 6E). Exercise did not alter erythrocyte count, WBC count, platelet count, PTH, or FGF23 levels (Supplemental Figure 6, F–H). These results indicate that light aerobic exercise protects against renal osteodystrophy in humans, without altering kidney function or whole blood count. We should acknowledge that our clinical data were preliminary, and the cohort size was limited. However, these results are consistent with findings from preclinical models and provide preliminary data that aerobic exercise have the potential for treating renal osteodystrophy.

### Irisin administration does not increase renal burden.

**Figure 7:**
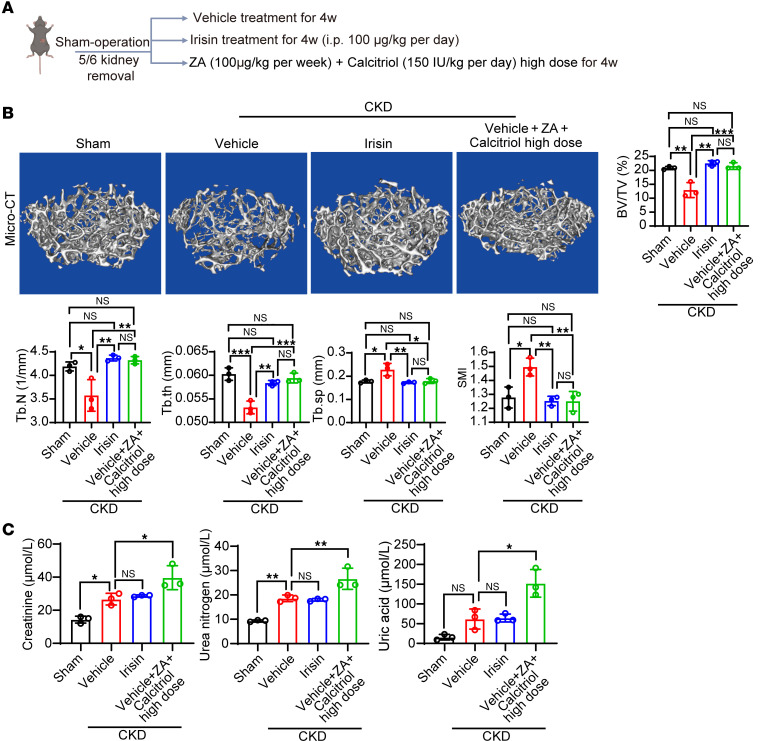
Irisin is not renal toxic for effective antiosteodystrophy treatment. (**A**) Schematic diagram of irisin administration or clinically used drug combinations including bisphosphonate (zoledronic acid) and calcium-absorbing drug (calcitriol) regimen in CKD model. (**B**) Representative μ-CT images of the femur tissues from CKD mice treated with vehicle, irisin, or high-dose drug combination. Sham-operated mice served as controls. Analysis of BV/TV, Tb.N, trabecular thickness (Tb.th), Tb.Sp, and SMI of these groups (*n* = 3 mice per group). (**C**) Serum creatinine, urea nitrogen, and uric acid levels in various groups of CKD mice or sham-operated mice (*n* = 3 mice per group). Data were analyzed by 1-way ANOVA (**B** and **C**). *P < 0.05; **P < 0.01; ***P < 0.001. Data were presented as mean ± SD.

Clinically, patients with renal osteodystrophy have limited medical options due to their severely impaired renal function. To assess the renal burden of irisin administration, we compared the nontreatment, clinically used drug combinations including bisphosphonate (zoledronic acid) and calcium-absorbing drug (calcitriol), and exogenous irisin in healthy mice (Supplemental Figure 7A) and CKD mice (Figure 7A). Interestingly, 4 weeks of clinically used drug combinations did not alter creatinine levels but increased urea nitrogen and uric acid in healthy mice (Supplemental Figure 7B), suggesting a modestly increased renal burden. Exogenous irisin administration did not change renal function markers in healthy mice (Supplemental Figure 7B). Next, we investigated the renal burden in CKD models. As expected, although both irisin and clinically used drug combinations improved bone quality to the same extent (Figure 7B), clinically used drug combinations significantly increased kidney burden in CKD mice, whereas irisin did not alter renal function markers (Figure 7C), suggesting that irisin administration is a kidney friendly, potent antirenal osteodystrophy monotherapy.

### Combination antiosteodystrophy therapy of irisin and clinically used antiresorptive drugs.

**Figure 8:**
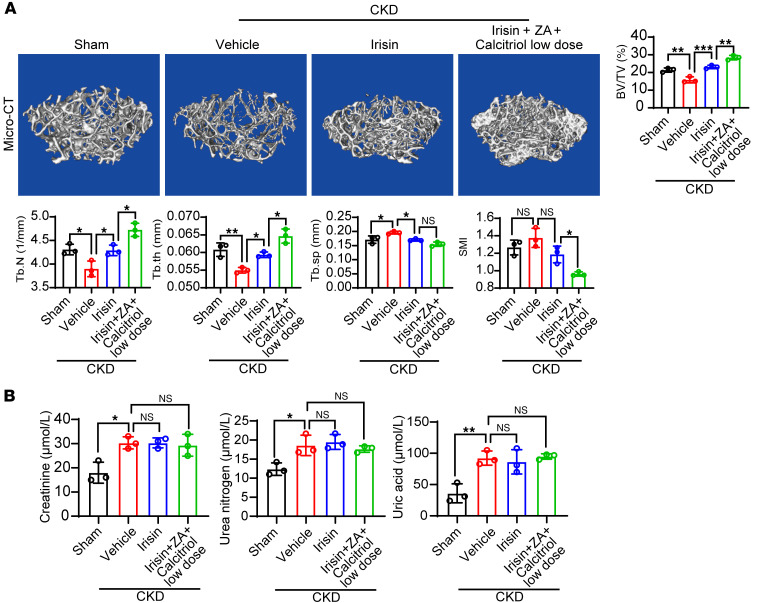
Irisin can be combined with conventional therapies for effective antiosteodystrophy treatment. (**A**) Representative μ-CT images of the femur tissues from vehicle-, irisin-, or combination therapy–treated (treated with irisin + low-dose drugs) CKD mice. Sham-operated mice served as controls. Analysis of BV/TV, Tb.N, trabecular thickness (Tb.th), Tb.Sp, and SMI of these groups (*n* = 3 mice per group). (**B**) Serum creatinine, urea nitrogen, and uric acid levels in various groups of CKD mice or sham-operated mice (*n* = 3 mice per group). Data were analyzed by 1-way ANOVA (**A** and **B**). *P < 0.05; **P < 0.01; ***P < 0.001. Data were presented as mean ± SD.

To investigate whether irisin can be used in combination with clinically used drugs, we chose a low dose of these drugs. This dosage is reported as a minimal effective dose (32, 33), approximately 20% of the full dose (Supplemental Figure 8A). As expected, this low dose did not exacerbate kidney burden (Supplemental Figure 8B), but unfortunately, it was ineffective in rescuing renal osteodystrophy in our CKD model (Supplemental Figure 8C). Next, we treated the mice with irisin or a combination of irisin and low-dose drugs. Surprisingly, the combination of irisin and low-dose drugs exhibited better antirenal osteodystrophy effects than irisin alone (Figure 8A). Importantly, similar to irisin alone, the combination therapy of irisin and low-dose drugs did not increase renal burden in CKD models (Figure 8B). These results support that irisin can be combined with current antiresorptive medications for the effective treatment of renal osteodystrophy.

## Discussion

Bone and mineral turnover disorders are common complications in patients with CKD, predisposing them to an increased risk of bone fractures (34). Furthermore, mineral turnover disorders further increase hyperparathyroidism or vascular calcification, which increases the risk of cardiovascular events (35). Within the broad spectrum of CKD-associated mineral and bone disorders (CKD-MBD), the diagnosis and management of renal osteodystrophy is particularly complex. BMD cannot distinguish among renal osteodystrophy, osteoporosis, and other metabolic bone diseases. Prior to the use of antiresorptive medications in CKD, it is important to evaluate CKD-MBD by measuring baseline serum calcium, phosphate, PTH, alkaline phosphatase, and 25-hydroxyvitamin D. For osteoporosis, various medication options, including the primary agent nitrogen-containing bisphosphonates, are effective and convenient. However, nephrotoxicity has limited their use in renal osteodystrophy. Hormones and monoclonal antibodies can be prescribed for renal osteodystrophy, but their high cost often limits their use (36, 37). Therefore, we would like to explore alternative methods to manage renal osteodystrophy without renal burden. In our study, we confirm that a moderate, clinically relevant aerobic exercise effectively alleviates renal osteodystrophy in mouse models and humans. Mechanistically, we have elucidated the PGC-1α/FNDC5/irisin axis in skeletal muscle and the direct proosteogenic role of irisin-integrin αvβ5 signaling on osteoblasts in renal osteodystrophy. Therapeutically, aerobic exercise or irisin administration can be used as a renal-friendly therapy for renal osteodystrophy, and it can be combined with conventional therapeutics to reduce therapy-related nephrotoxicity. We have conducted a pilot clinical study demonstrating that intradialysis aerobic exercise prevents the progression of renal osteodystrophy and increases serum irisin levels. There is limited evidence on the effect of exercise on bone parameters in patients with renal osteodystrophy. Reports have suggested either unaltered bone metabolism markers (38, 39) or improved bone density (40). The conflicting evidence highlights the need for mechanistic investigation of exercise therapy in patients with CKD-BMD. In contrast to the reports that found no benefit for bone metabolism, our study recruited patients without metabolic diseases and applied a longer-term exercise training plan. Perhaps physical activity has a mild supporting effect on bone density, which requires a relatively long-term process to take effect, and may be masked by other metabolic diseases that may affect bone metabolism.

Irisin is cleaved from FNDC5, which is expressed in muscle under the control of PGC-1α. Irisin is released into the circulation upon physical exercise and is capable of stimulating adipocyte tissue browning (14), reducing hepatic cholesterol synthesis (41), boosting memory (22), and increasing cortical bone mass (42). The pathophysiological function of irisin is under intense investigation, and its therapeutic potential has been proposed in various diseases, including osteoporosis. However, current treatment strategies for osteoporosis focus on mitigating osteoclast activity and increasing osteoblast function through medications like estrogen, bisphosphonates, denosumab, and calcitonin. Irisin is more likely to be used in the clinic to treat certain types of bone loss where therapeutic options are limited. In our work, irisin is administrated on a daily basis to mimic the daily aerobic exercise training plan in mice, which differs from the regimen in osteoporosis models where irisin was administered once a week (43–45). Although, among various dosages, a daily injection of 500 μg/kg irisin may be the optimal dose of efficacy in mice (46), we chose the lower dose of 100 μg/kg irisin per day to exclude the known *Ucp1*-promoting effect (47). Our work reveals the therapeutic potential of irisin in renal osteodystrophy and provides the conceptual basis for sustained irisin therapy and combination therapy of irisin and conventional antiresorptive drugs in the management of renal osteodystrophy.

In our CKD mouse model, we found that exercise not only improved renal osteodystrophy but also improved renal function to some extent. Interestingly, irisin exhibited an antiosteodystrophy effect without adding to the burden on the kidneys. Can exercise improve renal osteodystrophy or even improve renal function via an irisin-independent mechanism? This possibility warrants further investigation in patients with CKD. A recent report shows that irisin interacts with the type 2 TGFβ-1 receptor to improve kidney energy metabolism (48), suggesting that irisin may improve kidney function. However, we did not observe this in our model. Perhaps severe renal dysfunction cannot be rescued by improving kidney energy metabolism. Although our in vitro experiments demonstrated the direct regulatory effect of irisin on osteoblasts, we cannot dismiss the possibility that exercise may prevent renal osteodystrophy through irisin-independent pathways. It is reasonable to speculate that aerobic exercise, a low-cost, easy-to-achieve, noninvasive physiological therapy, may have even more profound benefits than irisin monotherapy in the management of renal osteodystrophy.

While we identified that the irisin/integrin αvβ5/osteoblast axis exists in renal osteodystrophy, the exact molecular mechanism underlying this antiosteodystrophy effect remains unclear. We noted that several pathways have been reported in other cell types or in non-CKD models. Our study focused on the translational aspect of exercise and irisin in treating renal osteodystrophy; the detailed molecular mechanism warrants future investigation. Furthermore, our study did not use irisin- or irisin receptor–specific KO mice to confirm the role of irisin in its antiosteodystrophy effect. Given the challenges associated with constructing such genetically modified animal models, we employed other approaches such as in vivo irisin supplementation and direct intervention on osteoblasts in vitro to strengthen our conclusions. Despite these potential limitations, to our knowledge, our study provides the first translational and clinical study of the role of exercise and irisin in patients with renal osteodystrophy, and it offers a practical therapeutic option for this population.

## Methods

### Sex as a biological variable.

Our study examined male mice, because male animals exhibited less variability in phenotype. Our study recruited both male and female patients to determine the effect of exercise on renal osteodystrophy.

### Animals.

Male C57BL/6 mice and male *Ucp1^–/–^* mice (stock no. T037633) in the C57BL/6 background between 6 and 8 weeks old were purchased from GemPharmatech and maintained under a 12-hour dark/12-hour light cycle with food and water provided ad libitum. All animals were randomly assigned to groups before experiments. The experimenter was not blind to group allocation and outcome assessment. No samples, animals, or data were excluded.

### Cell culture, cell differentiation, and cell sorting.

The murine preosteoblast cell line MC3T3-E1 subclone 14 was provided by Xiangzhong Zhao (the Affiliated Hospital of Qingdao University, Qingdao, China). MC3T3-E1 cells were cultured in 10% FBS-DMEM (40130ES76, YEASEN; TBD10569, TBD Science), containing 100 U/mL penicillin and 100 μg/mL streptomycin (MA0110, Meilunbio). All cell lines used in our study were negative for mycoplasma. Mature osteoblasts were induced using the MC3T3-E1 cell osteogenic differentiation basal medium (MUXMT-90021, Cyagen) for 7 days. For magnetic-activated cell sorting (MACS), fresh dissected femurs were smashed in ice-cold PBS and then digested in PBS containing 0.1% collagenase I and II (40507ES60, YEASEN; 40508ES60, YEASEN) at 37°C for 40–60 minutes with gentle pipetting. After digestion, cells were washed with ice-cold PBS and resuspended by 1 mL MACS buffer (a solution containing PBS, 0.5% BSA, and 2 mM EDTA) and were stained with a mouse anti–mouse/human RANK antibody (ab13918, Abcam), followed by an Alexa Fluor 647–conjugated donkey anti-mouse antibody (A31571, Invitrogen). Anti–Alexa Fluor 647 MicroBeads (130-091-395, Miltenyi Biotec) were subsequently used for magnetic labeling. After washing, positive and negative cells were sorted with a MACS column and magnetic MACS separators (130-042-201, Miltenyi Biotec; 130-042-303, Miltenyi Biotec) (49). For osteoclast-like cells, RANK^+^ population was harvested. For osteoblast-like cells, RANK^–^ population was stained again with a mouse anti–mouse/human CD45 antibody (17-0451-82, Invitrogen), and the RANK^–^CD45^–^ cell population was harvested.

### Animal models.

For CKD model, mice were anaesthetized by isoflurane (R510-22, RWD Life Science) using a small animal anesthesia system (ALC-Ane6-V, Alcott Biotech). Both kidneys were exposed, and nephrectomy was performed for left kidney and two-thirds of the right kidney. The wound was sutured using the sterile surgical suture (CR436, Jinhuan Medical), and the animals were kept at room temperature until recovery from the operation. Osteodystrophy was detected 5 weeks after nephrectomy. For aerobic exercise training, an 8-line electrical treadmill designed for rodents (ZH-PT/5S, Nengbowan) was used. Mice were acclimatized to the treadmill for 7 days at an increasing speed up to 12 m/min and a predetermined inclination of 10 degrees. After acclimatization, a typical light aerobic exercise program for mice was applied (20). All mice ran at a speed of 10 m/min for 15 minutes per day for 4 weeks. For BAT removal model, healthy mice were anaesthetized and a small incision was surgically created at the intrascapular area. Blood vessels were pinched to prevent excessive bleeding. BAT was carefully dissected, followed by the suturing of the incision.

### Drug administration.

For in vitro siRNA treatment, mouse *Itgb5* siRNA 5′-GGCCAGTTCTACACTACCA-3′ (siG151215105237, RIBBIO), and scrambled control siRNA were transfected into osteoblasts using an EZ Trans transfection reagent (AC04L051, Shanghai Life-iLab Biotech). Knockdown efficiency was confirmed after 24 hours of transfection by qPCR. For in vitro irisin treatment, Recombinant Human/Murine/Rat irisin (100-65-50, Peprotech) at 500 ng/mL was added into the cultured osteoblasts. For in vivo irisin treatment, mice were i.p. administrated with or without Recombinant Human/Murine/Rat irisin (100-65-50, Peprotech) at 100 μg/kg per day for 4 weeks. For in vivo drug treatment, mice were i.p. treated with 100 μg/kg of zoledronic acid (HY-13777, MedChemExpress) once per week and with 150 IU/kg per of calcitriol (17938, Sigma-Aldrich) once per day for high-dose antiosteodystrophy treatment. For low-dose antiosteodystrophy treatment, mice were treated with 20 μg/kg of zoledronic acid (HY-13777, MedChemExpress) once per week and with 25 IU/kg per of calcitriol (17938, Sigma-Aldrich) once per day. After experiment, animals were sacrificed at different time points. Fresh tissues were collected for further investigation.

### μ-CT analysis.

Mice were sacrificed and perfusion fixation was performed through the heart using 4% PFA. Bone tissues were fixed in 4% PFA for additional 2 days before being scanned by an ex vivo x-ray microtomography (Skyscan 1272, Bruker Micro CT). The growth plate slice was defined as 2D image 0. For bone scanning, the ROI was selected from 2D image 80 to image 180. Morphometric parameters and 3D reconstruction were analyzed using CTAn software (Bruker Micro CT). The 3D models were adjusted in CTVol software (Bruker Micro CT) (50).

### Histochemical staining.

For histological analysis, mouse femur bone tissues were decalcified in EDTA and embedded in paraffin. Paraffin-embedded tissues (5 μm thick) were prepared and mounted onto glass slides. Slides were baked for 1 hour at 60°C, deparaffinized in xylene (10023418, SCR), and sequentially rehydrated in 99%, 95%, and 70% ethanol (10009218, SCR). Tissue slides were counterstained with Haematoxylin (Mayer’s) (MB9897, Meilunbio) and Eosin (MA0164, Meilunbio) before dehydration with 95% and 99% ethanol, and were mounted with neutral balsam (1004160, SCR). Stained tissues were analyzed under a light microscope (Leica DM IL LED). For histochemistry, bone samples were stained with Safranin O and Fast Green staining kit (G1371, Solarbio). Slides were incubated in Fast Green for 5 minutes, washed with distilled water, and then incubated in Sirius Red for 10 seconds. Slides were then dehydrated in ethanol and mounted. For histomorphometry, bone samples were stained with TRAP staining kit (G1050, Servicebio) and counter stained with hematoxylin solution (G1004, Servicebio). Other samples were stained with Goldner staining kit (G1064, Servicebio). Differentiated osteoblasts were cultured on round coverslips and stained with ALP staining kit (BP090, Biossci) or Alizarin red S staining solution (C0140, Beyotime). Cell slides were fixed in 95% ethanol for 10 minutes,and incubated in ALP staining solution or 0.2% Alizarin red S Staining Solution (pH 8.3) for 30 minutes. Slides were then washed with distilled water and mounted.

### Calcein–Alizarin red S labeling.

Mice were i.p. injected with 20 mg/kg calcein (C0875-5G, MilliporeSigma, 1 mg/mL in 2% NaHCO_3_ solution) and 40 mg/kg Alizarin red S (A5533-25G, MilliporeSigma, 2 mg/mL in distilled water) on day 22 and day 25 of the 4-week irisin administration, and they were sacrificed at day 28. Femur bone samples were sectioned at 8 μm using a hard tissue cutter (HistoCore AUTOCUT, Leica) and examined using a digital scanner (Pannoramic 250FLASH, 3DHISTECH).

### Renal function test and electrolyte test in CKD mice.

Peripheral blood samples from mice were collected into anticoagulation tubes and were centrifuged at 3,300 g for 10 minutes to collect serum. Kidney function was analyzed by measuring serum levels of creatinine (OSR6178, Beckman Coulter), blood urea nitrogen (OSR6134, Beckman Coulter), uric acid (OSR6198, Beckman Coulter), calcium (OSR60117, Beckman Coulter), and phosphorous (OSR6122, Beckman Coulter) in clinical chemistry analyzers (AU5800, Beckman Coulter).

### ELISA.

Mouse blood samples were collected into anticoagulation tubes, and were centrifuged at 3,300 g for 10 minutes to collect serum. Mouse and human serum irisin were detected with commercially available ELISA kits (E-EL-M2743c, Elabscience; E-EL-M5735c, Elabscience) following the manufacturer’s recommendations. Mouse serum crosslaps and P1NP levels were detected with commercially available ELISA kits (JN80342, Jining Shiye Biotechnology; E-EL-M0233, Elabscience). Mouse and human serum FGF23 levels were detected with commercially available ELISA kits (E-EL-M2415, Elabscience; E-EL-H1116, Elabscience). Mouse serum PTH levels were detected with a commercially available ELISA kit (E-EL-M0709c, Elabscience). Absorbance values were detected at 450 nm using a Synergy 2 Multi-Mode Microplate Reader (BioTek). Human serum PTH levels were detected (A16972, Beckman Coulter) using clinical chemistry analyzers (AU5800, Beckman Coulter).

### Immunoblot.

Cultured cells were lyzed in a RIPA lysis buffer containing proteinase and phosphatase inhibitor cocktails (MA0151, Meilunbio; MB2678, Meilunbio; 1:100). An equal amount of protein samples from each group and a standard molecular weight marker (AP13L052, Life-iLab) were loaded on a 10% SDS-PAGE gel (AP15L945, Life-iLab), followed by transferring onto a PVDF membrane (IPVH00010, MilliporeSigma), which was subsequently blocked with 5% skimmed milk for 2 hours. Membranes were incubated overnight at 4°C with primary antibodies diluted in Primary Antibody Dilution Buffer (MB9881, Meilunbio). A rabbit anti-ALPL antibody (A0514, ABclonal; 1:1,000), a mouse anti–β-actin antibody (AC026, ABclonal; 1:1,000), a rabbit anti–PGC-1α antibody (A12348, ABclonal; 1:1,000), and a rabbit anti-FNDC5 antibody (AB174833, Abcam; 1:1,000) were used as primary antibodies. After rigorous washing with PBS containing 0.1% Tween-20 (T8220, Solarbio), membranes were incubated at room temperature for 2 hours with a goat anti–mouse HRP–conjugated IgG antibody (AS003, ABclonal; 1:5,000) or a goat anti–rabbit HRP–conjugated IgG antibody (AS014, ABclonal; 1:5,000). Target proteins were visualized via a super sensitive ECL luminescence reagent (MA0186, Meilunbio) with a Molecular Imager ChemiDoc XRS System (Bio-Rad).

### RNA extraction and qPCR.

Total RNAs were extracted from various tissues and cultured cells using a RNAsimple Total RNA kit (DP419, TIANGEN). Total RNA from each sample was reversely transcribed using a Hifair II 1^st^ Strand cDNA Synthesis SuperMix (11123ES60, YEASEN). Reverse transcription was performed at 42°C for 15 minutes and subsequently at 80°C for 5 minutes to inactivate the enzyme activity. The cDNA samples were subjected to qPCR using a StepOnePlus Real-Time PCR System (Applied Biosystems) (51). Each sample was triplicated and in a 10 μL reaction containing Hieff qPCR SYBR Green Master Mix (11203ES03, YEASEN), 50 nM forward and reverse primers, and 2 μL cDNA. The qPCR protocol was executed for 60 cycles, and each cycle consisted of denaturation at 95°C for 15 seconds, annealing at 60°C for 1 minute, and extension at 72°C for 1 minute. The primer pairs specific for various genes used in our experiments included: mouse *Ctsk* forward, 5′-GAAGAAGACTCACCAGAAGCAG-3′; mouse *Ctsk* reverse, 5′-TCCAGGTTATGGGCAGAGATT-3′; mouse *Actb* forward, 5′-GGCTGTATTCCCCTCCATCG-3′; mouse *Actb* reverse, 5′-CCAGTTGGTAACAATGCCATGT-3′; mouse *Alpl* forward, 5′-GGCACGTATGGCAGCAAGAT-3′; mouse *Alpl* reverse, 5′-CCAAGGAGGAGGATTCAAACTG-3′; mouse *Rankl* forward, 5′-CAGCATCGCTCTGTTCCTGTA-3′; mouse *Rankl* reverse, 5′-CTGCGTTTTCATGGAGTCTCA-3′; mouse *Adipor1* forward, 5′-TGTTCCTCTTAATCCTGCCCA-3′; mouse *Adipor1* reverse, 5′-CCAACCTGCACAAGTTCCCTT-3′; mouse *Bmp4* forward, 5′-TTCCTGGTAACCGAATGCTGA-3′; mouse *Bmp4* reverse, 5′-CCTGAATCTCGGCGACTTTTT-3′; mouse *Bglap* forward, 5′-TTCCCTGGGGAGGACTACTG-3′; mouse *Bglap* reverse, 5′-TGTATGCTTGCCCCGTGAAAT-3′; mouse *Tnfrsf11b* forward, 5′-ACCCAGAAACTGGTCATCAGC-3′; mouse *Tnfrsf11b* reverse, 5′-CTGCAATACACACACTCATCACT-3′; mouse *Mmp9* forward, 5′-CTGGACAGCCAGACACTAAAG-3′; mouse *Mmp9* reverse, 5′-CTCGCGGCAAGTCTTCAGAG-3′; mouse *Acp5* forward, 5′-CACTCCCACCCTGAGATTTGT-3′; mouse *Acp5* reverse, 5′-CATCGTCTGCACGGTTCTG-3′; mouse *Itgb5* forward, 5′-GGACCGTGGATTGCCAAAGT-3′; mouse *Itgb5* reverse, 5′-GAAGTGCCACCTCGTGTGAA-3′; mouse *Fndc5* forward, 5′-TTGCCATCTCTCAGCAGAAGA-3′; and mouse *Fndc5* reverse, 5′-GGCCTGCACATGGACGATA -3′.

### Exercise intervention for patients with CKD.

Exercise intervention was designed for patients with CKD following the recommendations (30). All volunteers were randomly assigned into 2 groups for moderate bedside bicycle exercise or resting. Volunteers in the exercise group underwent 12 weeks of intradialysis aerobic exercise using an adjustable horizontal treadmill attached to the bed. Patients in the supine position were assisted and performed lower limb activities by active pedaling action. Exercise was performed during dialysis, 3 times a week for approximately 35 minutes each time for 12 weeks. The exercise regimen consisted of passive exercise (0–10 watts) for 5–10 minutes, active pedaling exercise (15–40 watts) for 20–30 minutes, slow (0–10 watts) active/passive relaxation exercise for 5–10 minutes, and leg stretching for 3–5 minutes. Volunteers in the resting group received no intervention and remained in the supine position as much as possible. To avoid hemodynamic instability, bedside pedal cycling was performed within 30–120 minutes after dialysis began. Exercise should not be performed in the following conditions: systolic blood pressure > 180 mm Hg and/or diastolic blood pressure > 110 mm Hg; an increase in body mass of > 5% during the interdialytic period; difficulty in establishing of vascular access; and any symptom that may prevent exercise. Exercise should be stopped if the following symptoms occur: severe fatigue (Borg scale [a scoring system for rating of perceived exertion and monitoring progress and mode of exercise] score > 15); chest pain; hypoglycemia; dizziness; pallor; syncope; vasovagal reaction; dyspnea; cardiac arrhythmia; hypotensive or hypertensive response. During the study, participants were closely monitored for possible and unintended adverse events by the study team and an experienced care team.

### Data collection for patients with ESRD.

Demographic data were recorded at the time of enrollment, and the patients underwent BMD test by dual-energy x-ray absorptiometry (Excellus, OsteoSys) at weeks 0, 4, 8, and 12, and blood sampling at weeks 0, 2, 4, 8, and 12. Blood samples were collected after overnight fasting (≥10 hours) prior to hemodialysis. Complete blood counts were performed by an Auto Hematology Analyzer (BC-5380, Mindray). Blood calcium and phosphorus were detected by a Clinical Chemistry Analyzer (BS-2000, Mindray) using corresponding kits (IG16300 and IG18300, Neusoftmedical). Irisin concentration was detected using a human irisin ELISA kit (GR-E11710, Fanyin Biotech) according to the instructions.

### Statistics.

Statistical computations were performed using GraphPad Prism (GraphPad). Statistical differences between 2 groups were determined by a 2-tailed Student’s *t* test. *P* < 0.05 was considered statistically significant. Differences among multiple groups were evaluated using 1-way ANOVA, as appropriate. Data are presented as mean ± SD.

### Study approval.

All animal studies were approved by the Animal Experimental Ethical Committee of the Fudan University, Shanghai, China (no. 20220228-098). The human study was approved by the Research Ethics Committee of the First Hospital of Longyan City, Fujian Province, China (no. LYREC2023-011-01) and recruited Asian Han Chinese individuals. The protocol was in accordance with the Declaration of Helsinki, and written informed consent was obtained from all volunteers before the start of the experiment.

### Data availability.

All the data are available from the corresponding author upon request. Values for all data points in graphs are reported in the Supporting Data Values file.

## Author contributions

MW, SX, XS, and Y Ye generated the ideas and designed experiments. MW, HL, LC, YW, WC, YT, ML, and JD performed clinical studies. XS, RC, MM, KR, ZP, Y Yang, SZ, QR, SX, and Y Ye performed preclinical studies. MW, HL, XS, RZ, MR, SX, Y Ye, and JW participated in discussions. RZ, MR, MW, SX, and JW provided important materials and reagents. SX and Y Ye wrote the manuscript. All authors approved the final version of the manuscript. MW, HL, and XS are listed as co–first authors. This order is because MW led the clinical studies, HL performed the clinical studies, and XS performed some of the preclinical studies.

## Supplementary Material

Supplemental data

Unedited blot and gel images

Supporting data values

## Version 1 01/30/2025

In-Press Preview

## Version 2 03/10/2025

Electronic publication
