# Supplementary material for: Aerobic exercise prevents renal osteodystrophy via irisin-activated osteoblasts
Source: JCI Insight. 2025 Jan 30;10(5):e184468. doi: 10.1172/jci.insight.184468 (PMC11949034; doi:10.1172/jci.insight.184468)
Supplement: Supplemental data [file jciinsight-10-184468-s083.pdf]

Aerobic exercise prevents renal osteodystrophy via irisin-activated osteoblasts

Meng Wu[1,2#], Huilan Li[2,3#], Xiaoting Sun[4#], Rongrong Zhong[5], Linli Cai[5], Ruibo Chen[6], Madiya Madeniyet[6], Kana Ren[6], Zhen Peng[6], Yujie Yang[7], Weiqin Chen[2], Yanling Tu[2], Miaoxin Lai[2], Jinxiu Deng[2], Yuting Wu[5], Shumin Zhao[5], Qingyan Ruan[5], Mei Rao[5], Sisi Xie[5]*, Ying Ye[8]*, Jianxin Wan[1,9,10]*

[1]Department of Nephrology, Blood Purification Research Center, the First Affiliated Hospital, Fujian Medical University, Fuzhou, China.

[2]Department of Nephrology, Longyan First Affiliated Hospital of Fujian Medical University, Longyan, China

[3]Department of Nephrology, Xuanwu Hospital, Capital Medical University, Beijing, China

[4]School of Pharmaceutical Science, Wenzhou Medical University, Wenzhou, China

[5]Department of Cardiology, Basic scientific research center, Longyan First Affiliated Hospital of Fujian Medical University, Longyan, China

[6]Department of Cellular and Genetic Medicine, School of Basic Medical Sciences, Fudan University, Shanghai, China

[7]Fundamental Research Center, Shanghai Yangzhi Rehabilitation Hospital (Shanghai Sunshine Rehabilitation Center), School of Medicine, Tongji University, Shanghai, China.

[8]Department of Oral Implantology, Stomatological Hospital and Dental School of Tongji University, Shanghai Engineering Research Center of Tooth Restoration and Regeneration, Shanghai, China.

[9]Fujian Clinical Research Center for Metabolic Chronic Kidney Disease, the First Affiliated Hospital, Fujian Medical University, Fuzhou, China.

[10]Department of Nephrology, National Regional Medical Center, Binhai Campus of the First Affiliated Hospital, Fujian Medical University, Fuzhou, China.

[#]**These authors contributed equally.**

Running title: Exercise prevents renal osteodystrophy via irisin

***Corresponding authors**

Sisi Xie, Ph.D., 18111010026@fudan.edu.cn
Mobile: +86-18559305640
Department of Cardiology, Basic scientific research center, Longyan First Affiliated Hospital of Fujian Medical University, Longyan, 364000, China
ORCID: https://orcid.org/0000-0003-4923-5305

Ying Ye, M.D., Ph.D., ying.ye@tongji.edu.cn
Mobile: +86-15900504029
Department of Oral Implantology, Stomatological Hospital and Dental School of Tongji University, Shanghai Engineering Research Center of Tooth Restoration and Regeneration, Shanghai 200072, China.
ORCID: https://orcid.org/0000-0001-9722-3312

Jianxin Wan, M.D., Ph.D., wanjx@fjmu.edu.cn
Mobile: +86-13805052715
Department of Nephrology, Blood Purification Research Center, the First Affiliated Hospital, Fujian Medical University, Chazhong Road 20, Fuzhou, 350005, China
ORCID: https://orcid.org/0000-0002-6733-0472

Supplemental Figures and Figure Legends

Supplemental Figure 1

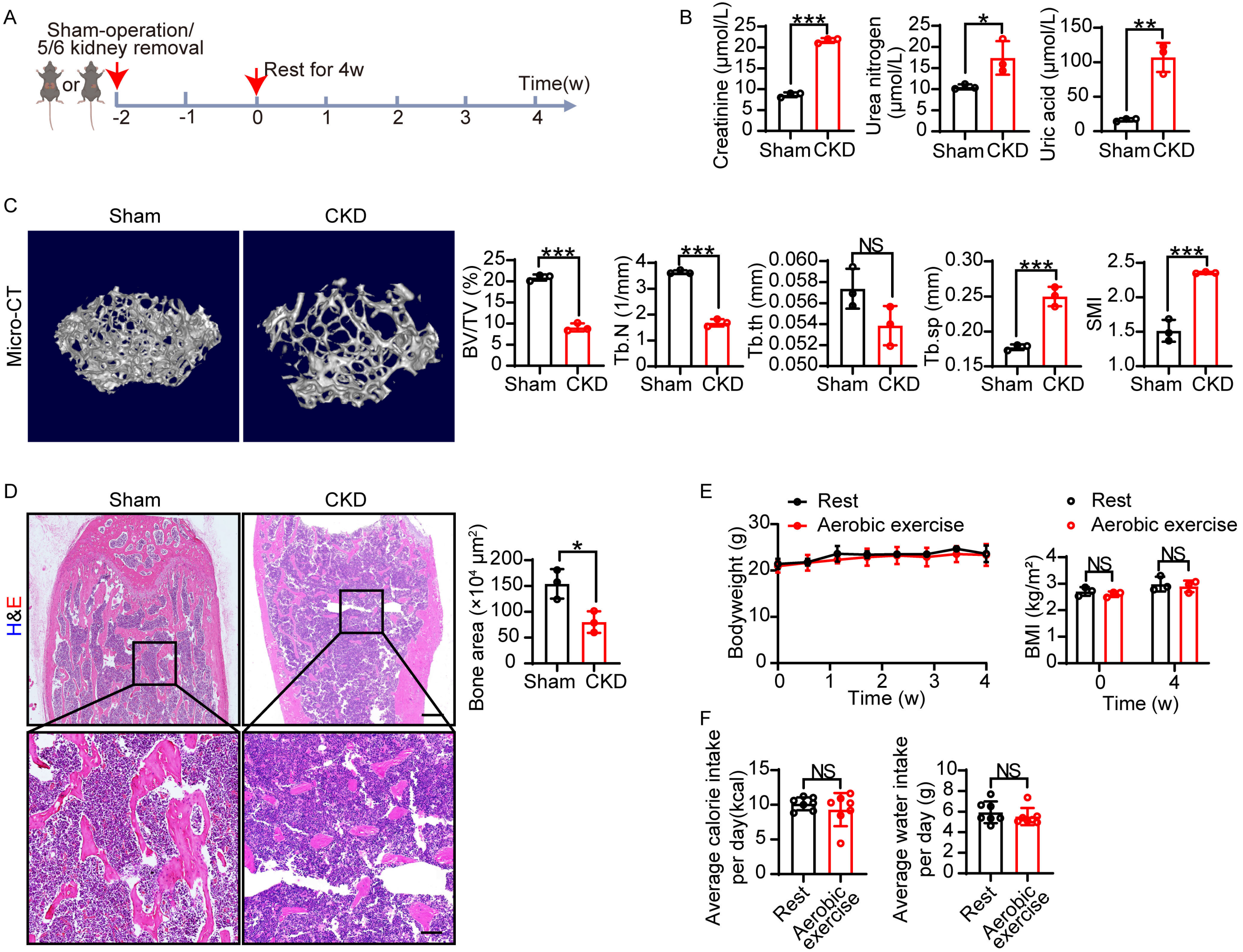

Figure S1. Modelling renal osteodystrophy by 5/6 nephrectomy in mice

(A) Schematic diagram of the CKD model establishment. Nephrectomy of left kidney and 5/6 of right kidney was performed for CKD modeling. Sham-operated mice were used as controls. One week after surgery, exercise training was performed by 1 week of adaptation and 4 weeks of treadmill aerobic exercise. **(B)** 6 weeks after surgery, serum creatinine, urea nitrogen, and uric acid levels in CKD mice or sham-operated mice (*n* = 3 mice per group). **(C)** Representative micro-CT images of the femur tissues from CKD mice or sham-operated mice. Analysis of total bone volume fraction (BV/TV), trabecular number (Tb.N), trabecular thickness (Tb.th), trabecular spacing (Tb.sp), and structure model index (SMI) of these groups (*n* = 3 mice per group). **(D)** Representative H&E staining images of the femur tissues from CKD mice or sham-operated mice. Scale bar in upper panel = 500 μm. Scale bar in lower panel = 100 μm. Quantification of the bone area (*n* = 3 mice per group). **(E)** Quantifications of bodyweight in resting or exercise-trained CKD mice (*n* = 3 mice per group). **(F)** Resting or exercise-trained CKD mice were measured for 7 days and the average caloric intake and water intake were quantified (*n* = 7 days per group). Data were analyzed by unpaired, 2-tailed Student's *t* test (**B, C, D, and F**) and two-way ANOVA (**E**). *p<0.05; **p<0.01; ***p<0.001. NS = not significant. Data were presented as mean ± s.d..

Supplemental Figure 2

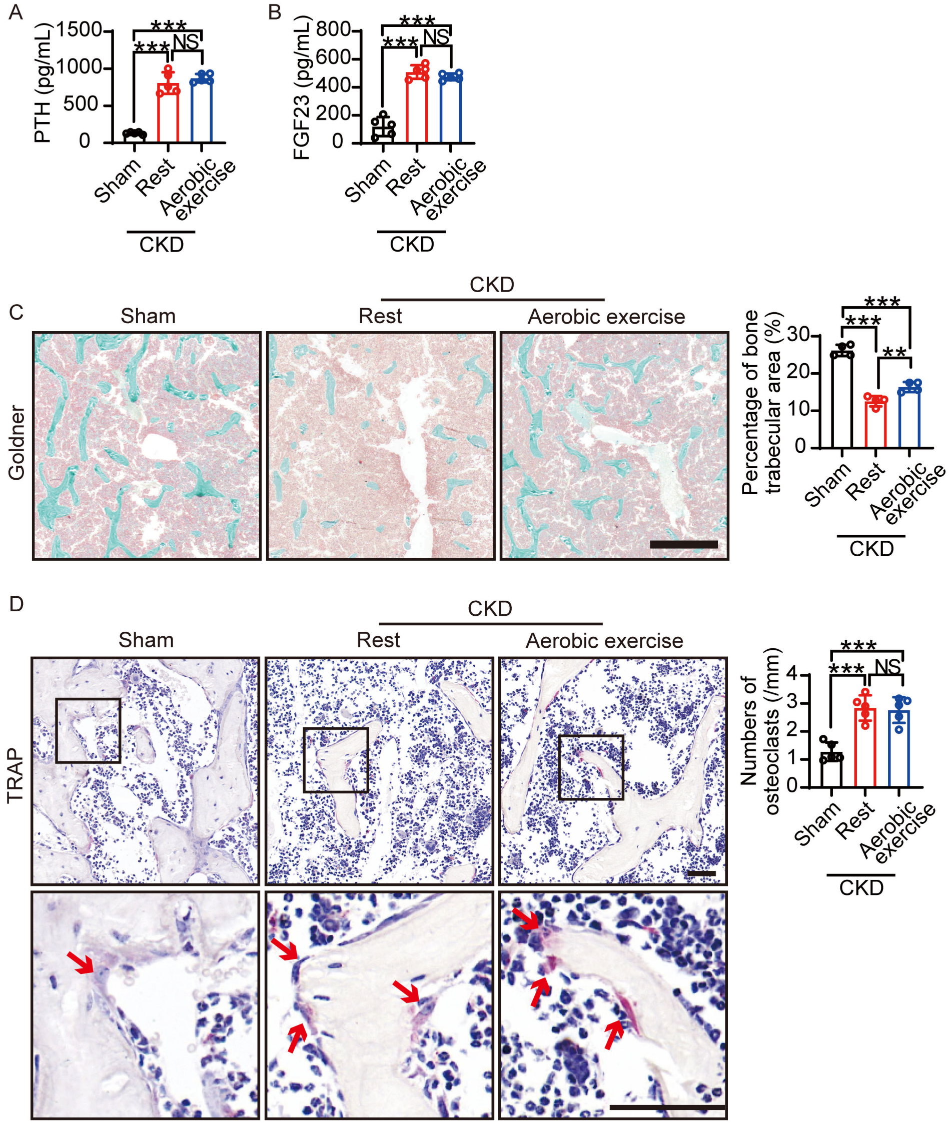

Figure S2. Exercise insignificantly alters renal osteodystrophy subtype

(A) Serum PTH levels from resting or exercise-trained CKD mice. Sham-operated mice were used as controls (*n* = 5 mice per group). **(B)** Serum FGF23 levels from resting or exercise-trained CKD mice. Sham-operated mice were used as controls (*n* = 5 mice per group). **(C)** Representative Goldner's staining images of the femur tissues from resting or exercise-trained CKD mice. Sham-operated mice served as controls. Scale bar = 500 μm. Quantification of percentages of staining positive bone trabecular areas (*n* = 5 mice per group). **(D)** Representative Tarp staining images of the femur tissues from resting or exercise-trained CKD mice. Sham-operated mice served as controls. Scale bar in upper panel = 50 μm. Scale bar in lower panel = 50 μm. Quantification of osteoclast numbers (*n* = 5 mice per group). Data were analyzed by one-way ANOVA (**A-D**). *p<0.05; **p<0.01; ***p<0.001. NS = not significant. Data were presented as mean ± s.d..

Supplemental Figure 3

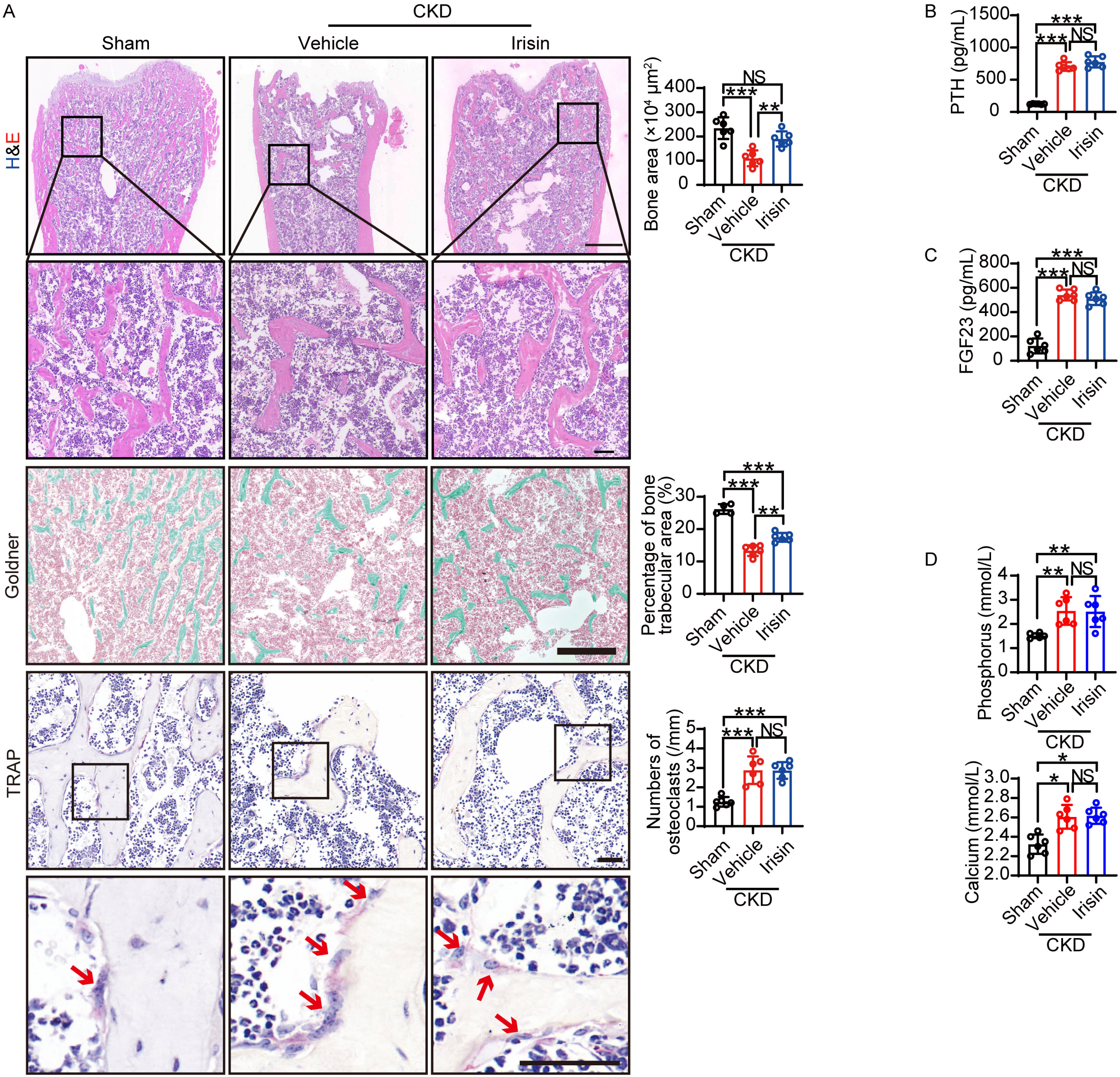

Figure S3. Irisin administration prevents renal osteodystrophy in mice

(A) Representative H&E staining images (Scale bar in upper panel = 500 μm. Scale bar in lower panel = 100 μm), Goldner's staining images (Scale bar = 500 μm), and Trap staining images Scale bar in upper panel = 50 μm. Scale bar in lower panel = 0 μm) of the femur tissues from vehicle- or irisin-treated CKD mice. Sham-operated mice served as controls. Quantification of the bone area, percentages of staining positive bone trabecular areas, and osteoclast numbers (*n* = 6 mice per group). **(B-D)** PTH, FGF23, calcium, and phosphorous in serum from vehicle- or irisin-treated CKD mice. Sham-operated mice served as controls (*n* = 6 mice per group). Data were analyzed by one-way ANOVA (**A-D**). *p<0.05; **p<0.01; ***p<0.001. NS = not significant. Data were presented as mean ± s.d..

Supplemental Figure 4

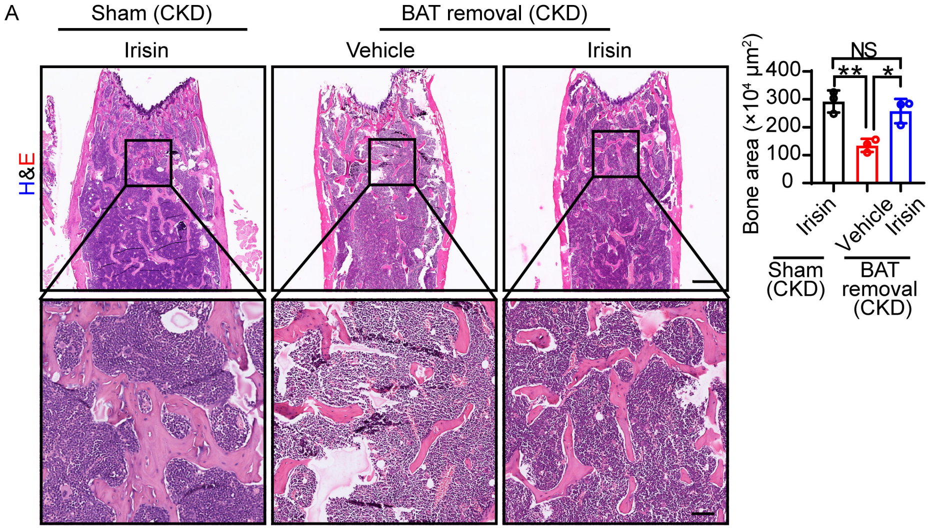

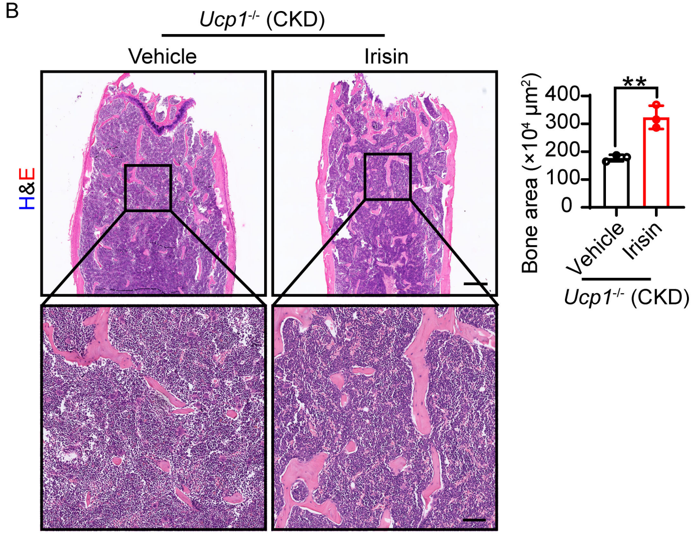

Figure S4. Irisin-induced anti-osteodystrophy effect in BAT removal mice and *Ucp1$^{-/-}$* mice

(A) Representative H&E staining images of the femur tissues from vehicle- or irisin-treated BAT-removed CKD mice. Irisin-treated sham-operated CKD mice served as controls. Scale bar in upper panel = 500 μm. Scale bar in lower panel = 100 μm. Quantification of the bone area (*n* = 3 mice per group). **(B)** Representative H&E staining images of the femur tissues from wildtype or *Ucp1$^{-/-}$* CKD mice. Scale bar in upper panel = 500 μm. Scale bar in lower panel = 100 μm. Quantification of the bone area (*n* = 3 mice per group). Data were analyzed by one-way ANOVA (**A**) and unpaired, 2-tailed Student's *t* test (**B**). *p<0.05; **p<0.01; ***p<0.001. NS = not significant. Data were presented as mean ± s.d..

Supplemental Figure 5

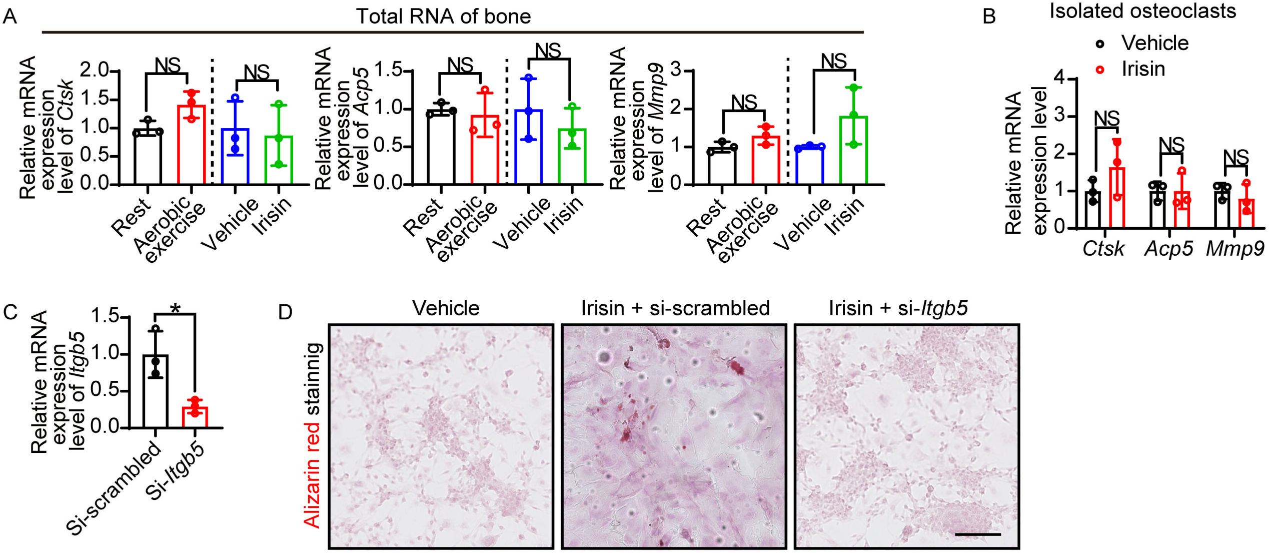

Figure S5. Irisin insignificantly affects osteoclasts

(A) *Ctsk*, *Acp5*, and *Mmp9* RNA expression levels in femur bone tissues from various groups (n = 3 mice per group). **(B)** *Ctsk*, *Acp5*, and *Mmp9* RNA expression levels in $RANK^+$ osteoclast cell populations isolated from femur bone tissues of vehicle- or irisin-treated CKD mice (n = 3 mice per group). **(C)** *Itgb5* RNA expression levels in MC3T3-E1 differentiated osteoblasts receiving si-scrambled or si-*Itgb5* pretreatment. (n = 3 mice per group). **(D)** Representative Alizarin Red staining images of irisin administrated MC3T3-E1 differentiated osteoblasts pretreated with si-scrambled or si-*Itgb5*. Data were analyzed by unpaired, 2-tailed Student's *t* test (**A-C**). *p<0.05; **p<0.01; ***p<0.001. NS = not significant. Data were presented as mean ± s.d..

Supplemental Figure 6

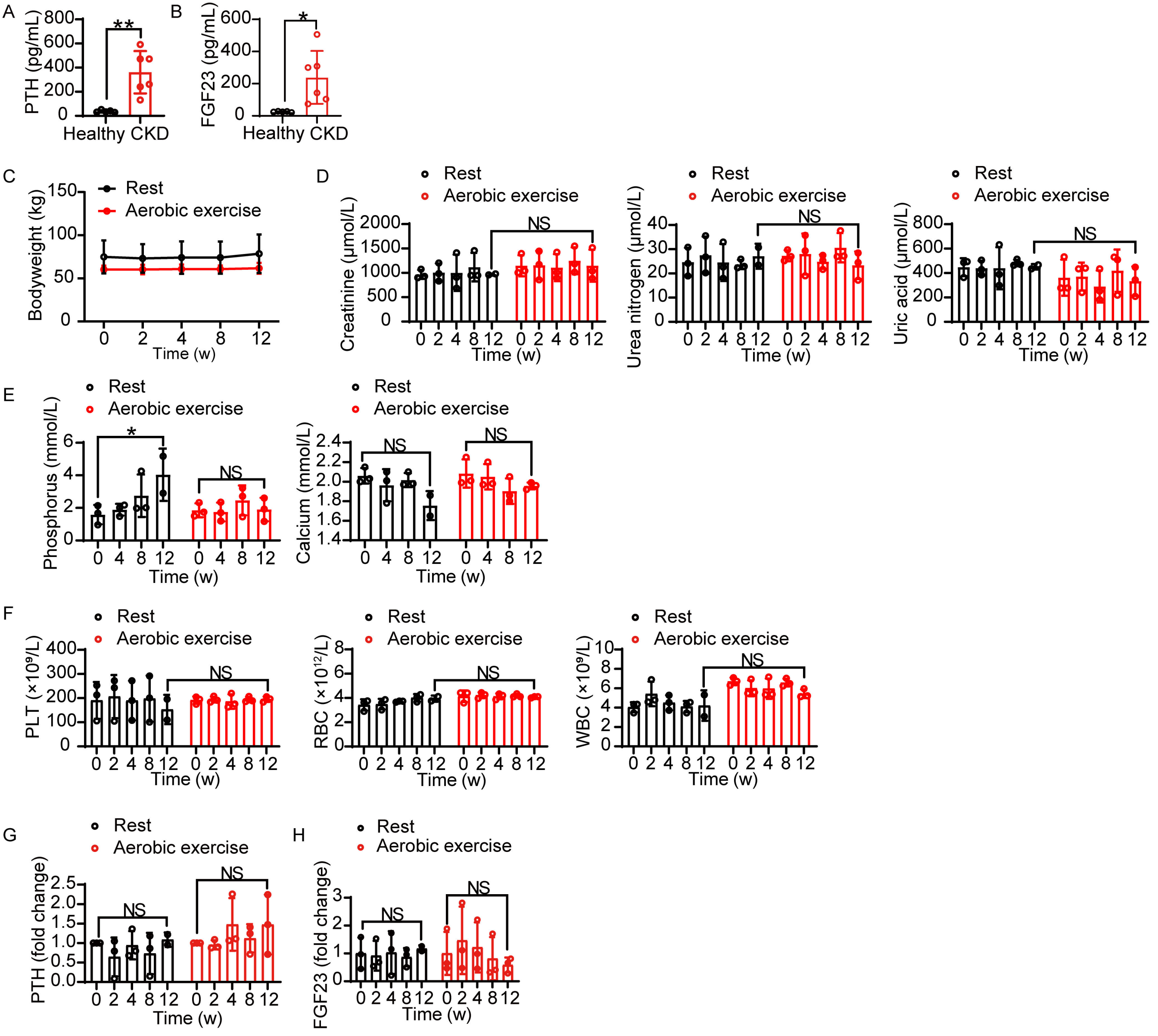

Figure S6. Aerobic exercise is a physical therapy without renal burden in ESRD patients

(A and B) Serum PTH levels and FGF23 levels from healthy volunteers or ESRD patients (*n* = 5 healthy volunteers and 6 patients). **(C)** Quantifications of bodyweight in resting or exercise-trained patients at week 0, 4, 8, 12 (*n* = 3 patients per group). **(D)** Serum creatinine, urea nitrogen, and uric acid levels in resting or exercise-trained patients at week 0, 2, 4, 8, 12 (*n* = 3 patients per group). Quantifications of bodyweight in resting or exercise-trained patients at week 0, 2, 4, 8, 12 (*n* = 3 patients per group). **(E)** Serum calcium and phosphorous in resting or exercise-trained patients at week 0, 4, 8, 12 (*n* = 3 patients per group). **(F)** Platelet counts (PLT), red blood cell counts (RBC), and white blood cell counts (WBC) in resting or exercise-trained patients at week 0, 2, 4, 8, 12 (*n* = 3 patients per group). **(G and H)** Relative changes of serum PTH levels and FGF23 levels in resting or exercise-trained patients at week 0, 2, 4, 8, 12 (*n* = 3 patients per group). Data were analyzed by unpaired, 2-tailed Student's *t* test (**A and B**) and two-way ANOVA (**D-G**). *p<0.05; **p<0.01; ***p<0.001. NS = not significant. Data were presented as mean ± s.d..

Supplemental Figure 7

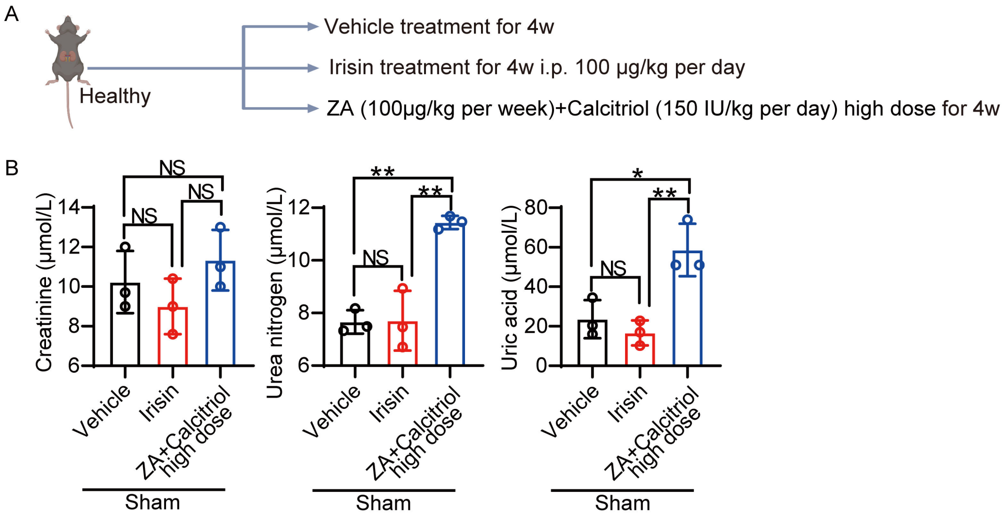

Figure S7. Renal toxicity of irisin and conventional anti-resorptive drugs in healthy mice

(A) Schematic diagram of irisin administration and clinically used drug combinations including bisphosphonate (zoledronic acid) and calcium-absorbing drug (calcitriol) regimen in healthy mice. **(B)** Serum creatinine, urea nitrogen, and uric acid levels in various groups of healthy mice (*n* = 3 mice per group). Data were analyzed by one-way ANOVA (**B**). *p<0.05; **p<0.01; ***p<0.001. NS = not significant. Data were presented as mean ± s.d..

Supplemental Figure 8

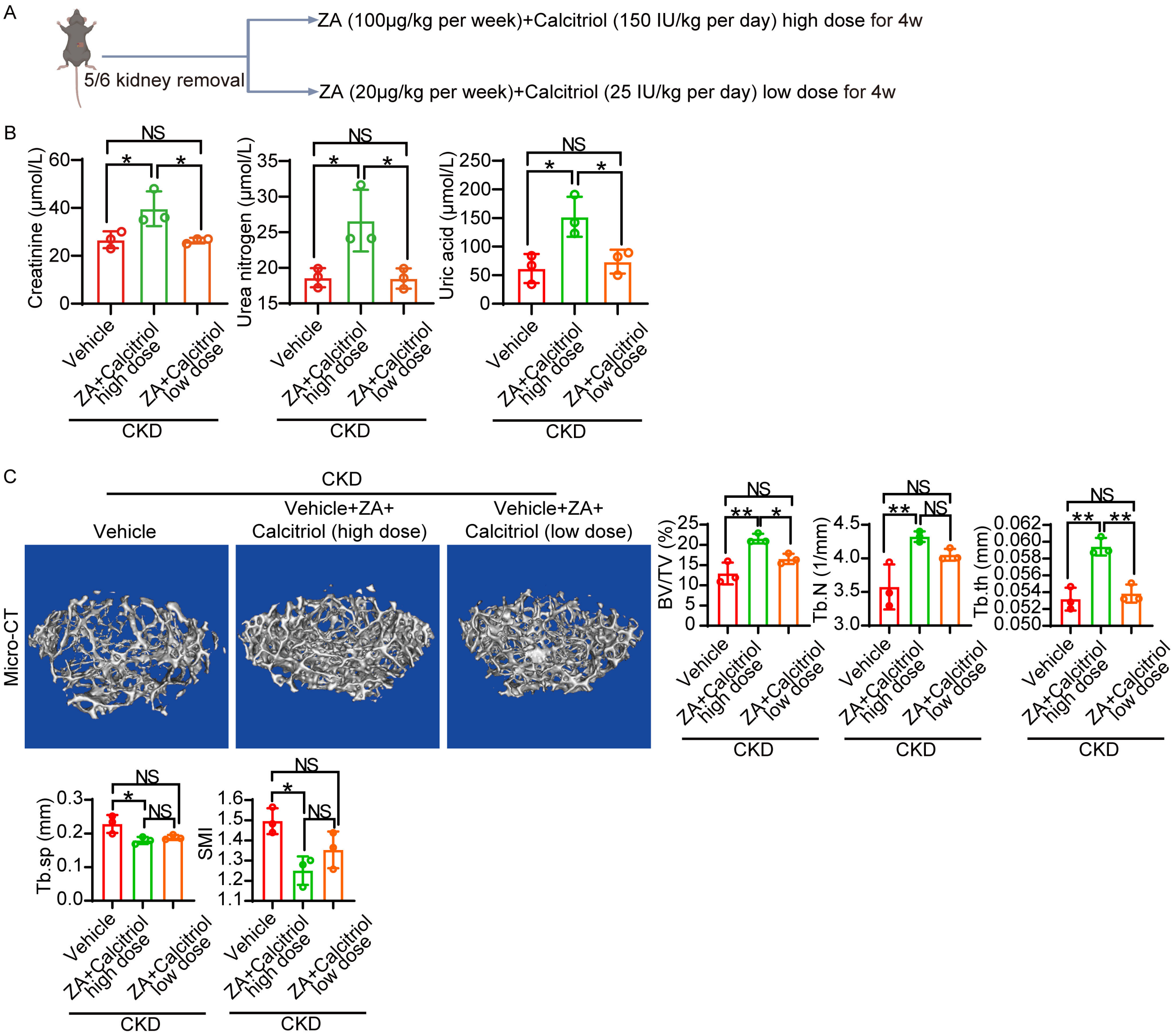

Figure S8. Renal toxicity of different doses of conventional anti-resorptive drugs in CKD mice

(A) Schematic diagram of high and low doses of clinically used drug combinations including bisphosphonate (zoledronic acid) and calcium-absorbing drug (calcitriol) regimen in CKD mice. **(B)** Serum creatinine, urea nitrogen, and uric acid levels in various groups of CKD mice (n = 3 mice per group). Data of vehicle- and high-dose drugs-treated groups were the same as in Figure 7, and served as controls. **(C)** Representative micro-CT images of the femur tissues from vehicle-, high-dose drugs-, or low-dose drugs-treated CKD mice. Analysis of total bone volume fraction (BV/TV), trabecular number (Tb.N), trabecular thickness (Tb.th), trabecular spacing (Tb.sp), and structure model index (SMI) of these groups (n = 3 mice per group). Data of vehicle- and high-dose drugs-treated groups were the same as in Figure 7, and served as controls. Data were analyzed by one-way ANOVA (**B and C**). *$p<0.05$; **$p<0.01$; ***$p<0.001$. NS = not significant. Data were presented as mean ± s.d..

Supplemental Tables

Table S1. Clinical data of ESRD patients and healthy volunteers

Number	Gender	Age	Diagnosis	CKD stage	Group
**1**	Male	57	Chronic kidney disease	5	Rest
**2**	Female	44	Chronic kidney disease	5	Rest
**3**	Male	61	Chronic kidney disease	5	Rest
**4**	Male	51	Chronic kidney disease	5	Aerobic exercise
**5**	Male	51	Chronic kidney disease	5	Aerobic exercise
**6**	Female	58	Chronic kidney disease	5	Aerobic exercise
**1**	Female	51	Healthy, no hypertension, no diabetes	N/A	N/A
**2**	Female	59	Healthy, no hypertension, no diabetes	N/A	N/A
**3**	Male	51	Healthy, no hypertension, no diabetes	N/A	N/A
**4**	Male	57	Healthy, no hypertension, no diabetes	N/A	N/A
**5**	Male	55	Healthy, no hypertension, no diabetes	N/A	N/A
