# Supplementary material for: Aerobic exercise prevents renal osteodystrophy via irisin-activated osteoblasts
Source: JCI Insight. 2025 Jan 30;10(5):e184468. doi: 10.1172/jci.insight.184468 (PMC11949034; doi:10.1172/jci.insight.184468)

Full unedited gel for Figure 2D

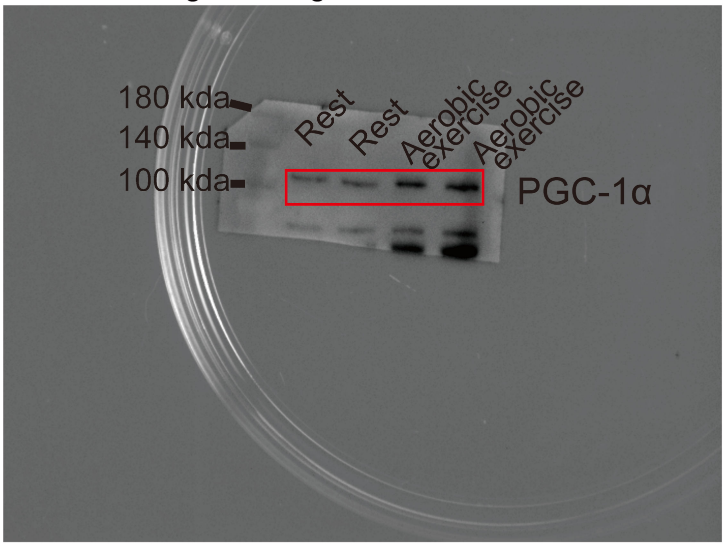

Full unedited gel for Figure 2D

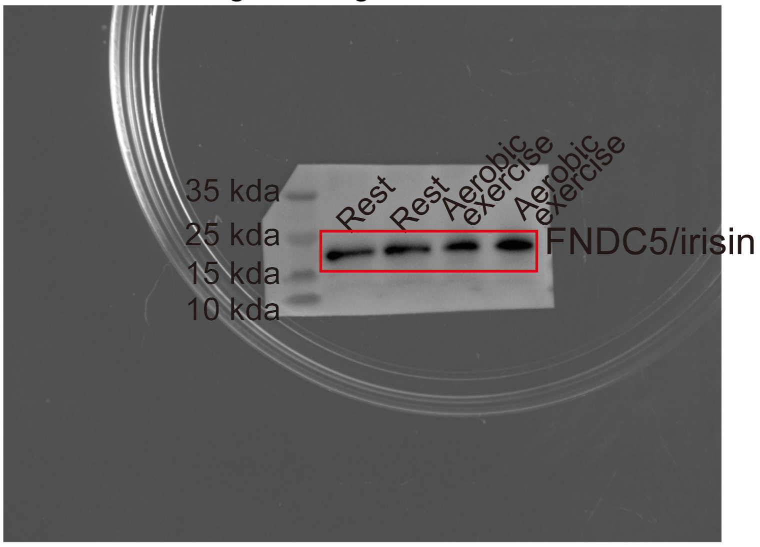

Full unedited gel for Figure 2D

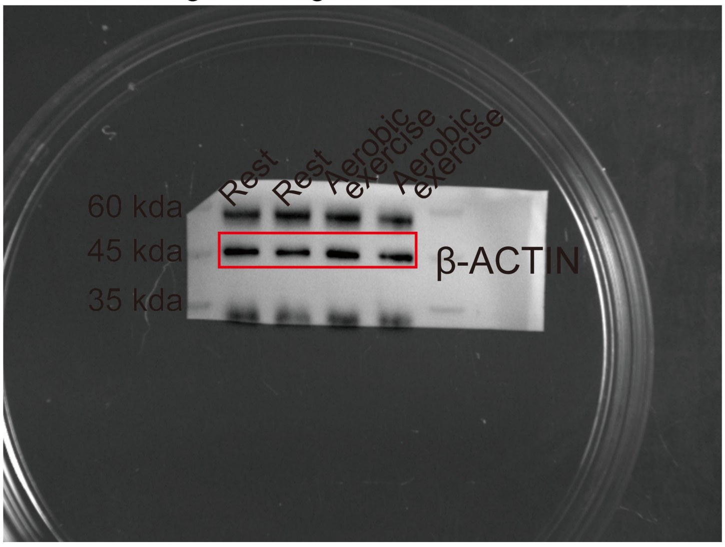

Full unedited gel for Figure 5C

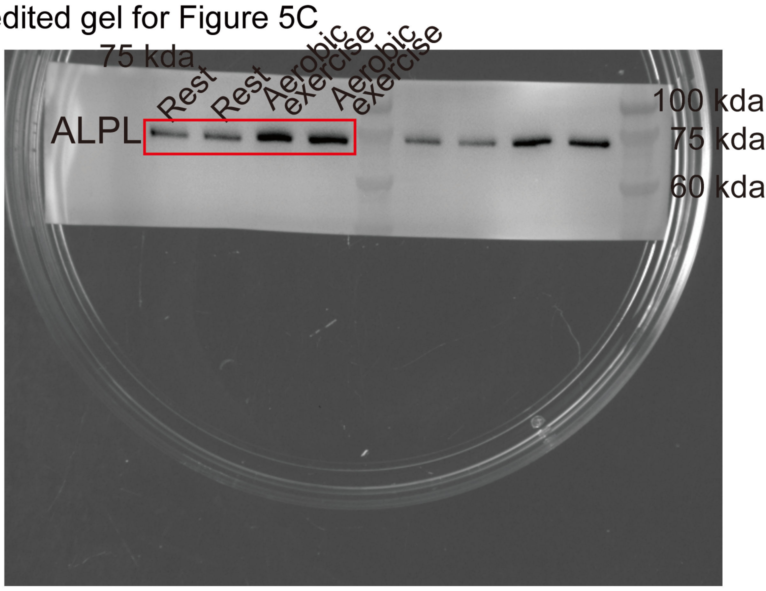

Full unedited gel for Figure 5C

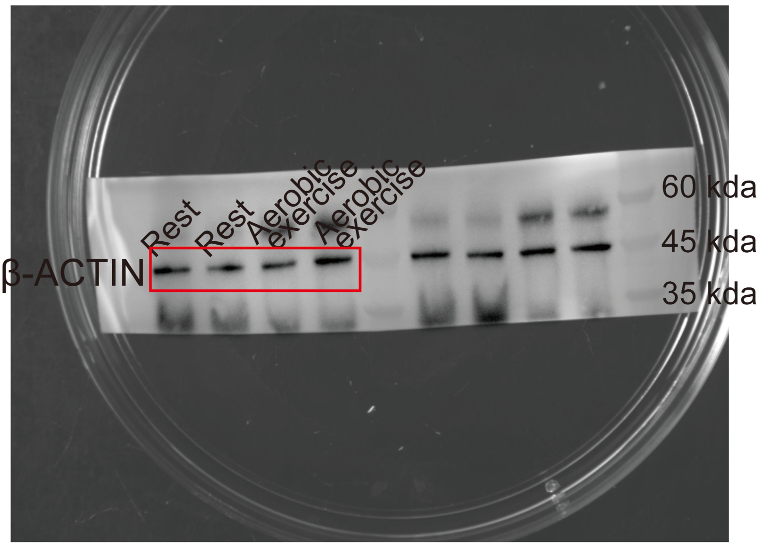

Full unedited gel for Figure 5C

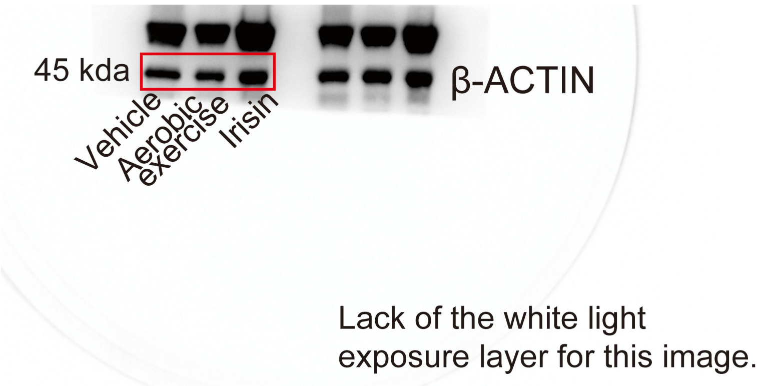

Full unedited gel for Figure 5C

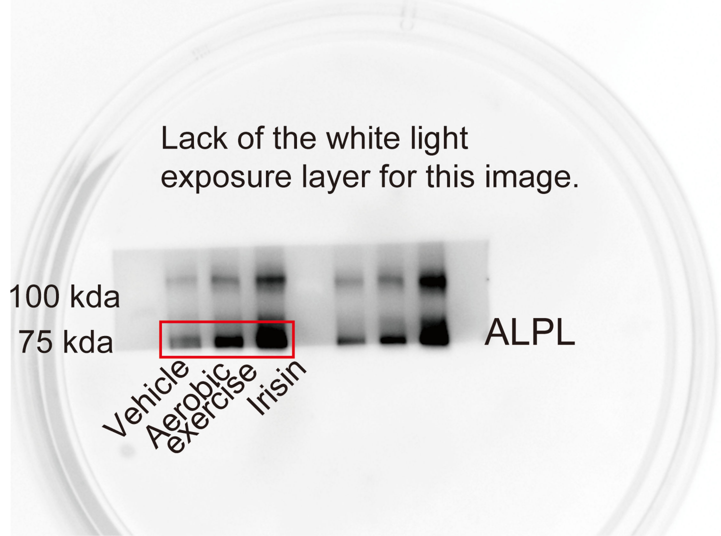

Supplement: Unedited blot and gel images [file jciinsight-10-184468-s084.pdf]
